# How the initiating ribosome copes with ppGpp to translate mRNAs

**DOI:** 10.1371/journal.pbio.3000593

**Published:** 2020-01-29

**Authors:** Daria S. Vinogradova, Victor Zegarra, Elena Maksimova, Jose Alberto Nakamoto, Pavel Kasatsky, Alena Paleskava, Andrey L. Konevega, Pohl Milón

**Affiliations:** 1 Petersburg Nuclear Physics Institute named by B.P. Konstantinov of NRC “Kurchatov Institute”, Gatchina, Russia; 2 NanoTemper Technologies Rus, Saint Petersburg, Russia; 3 Centre for Research and Innovation, Faculty of Health Sciences, Universidad Peruana de Ciencias Aplicadas (UPC), Lima, Peru; 4 Peter the Great Saint Petersburg Polytechnic University, Saint Petersburg, Russia; 5 NRC “Kurchatov Institute,” Moscow, Russia; Yale University, UNITED STATES

## Abstract

During host colonization, bacteria use the alarmones (p)ppGpp to reshape their proteome by acting pleiotropically on DNA, RNA, and protein synthesis. Here, we elucidate how the initiating ribosome senses the cellular pool of guanosine nucleotides and regulates the progression towards protein synthesis. Our results show that the affinity of guanosine triphosphate (GTP) and the inhibitory concentration of ppGpp for the 30S-bound initiation factor IF2 vary depending on the programmed mRNA. The *Tuf*A mRNA enhanced GTP affinity for 30S complexes, resulting in improved ppGpp tolerance and allowing efficient protein synthesis. Conversely, the *Inf*A mRNA allowed ppGpp to compete with GTP for IF2, thus stalling 30S complexes. Structural modeling and biochemical analysis of the *Tuf*A mRNA unveiled a structured enhancer of translation initiation (SETI) composed of two consecutive hairpins proximal to the translation initiation region (TIR) that largely account for ppGpp tolerance under physiological concentrations of guanosine nucleotides. Furthermore, our results show that the mechanism enhancing ppGpp tolerance is not restricted to the *Tuf*A mRNA, as similar ppGpp tolerance was found for the SETI-containing *Rnr* mRNA. Finally, we show that IF2 can use pppGpp to promote the formation of 30S initiation complexes (ICs), albeit requiring higher factor concentration and resulting in slower transitions to translation elongation. Altogether, our data unveil a novel regulatory mechanism at the onset of protein synthesis that tolerates physiological concentrations of ppGpp and that bacteria can exploit to modulate their proteome as a function of the nutritional shift happening during stringent response and infection.

## Abstract 2

During host colonization, the alarmones ppGpp and pppGpp reshape the bacterial proteome by acting on DNA, RNA, and protein synthesis. This study shows that inhibition of translation by ppGpp involves prevention of 30S initiation complex formation by ppGpp-bound IF2, with the extent of ppGpp tolerance depending on the specific mRNA.

## Introduction

**Fig 1:**
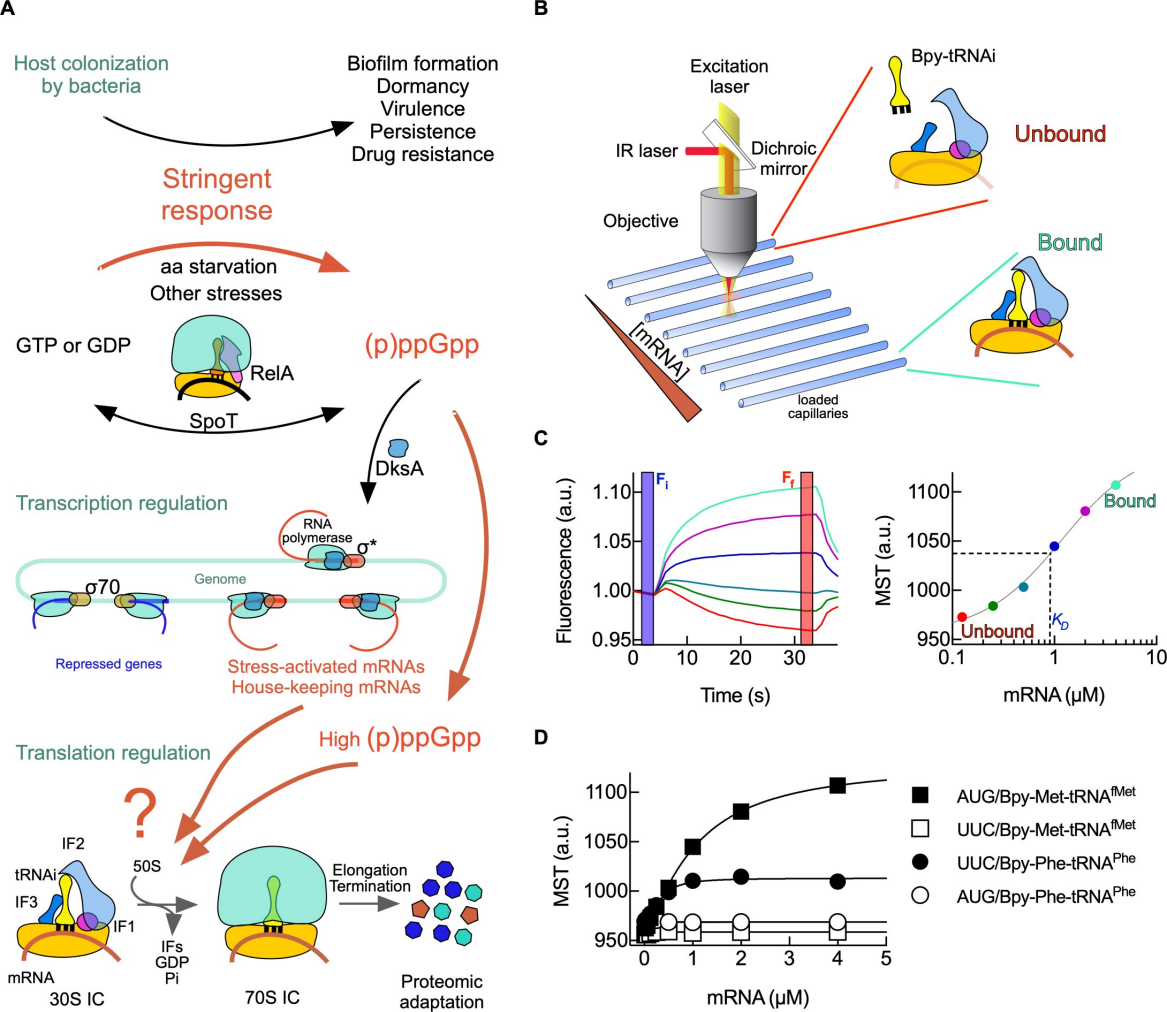
Bacterial stringent response and experimental approach. (A) Schematics representing the onset of stringent response, (p)ppGpp accumulation, and gene expression regulation. Translation of stress-related mRNAs upon (p)ppGpp accumulating is the focus of the investigation (indicated as the red question mark). (B) Schematics of instrumentation for measuring 30S initiation complex (IC) by fluorescence Microscale Thermophoresis (MST) (provided by Nanotemper Technologies). Generally, each capillary was loaded with 1 μM 30S subunits, 4 μM mRNA, 2 μM IF1, 1 μM IF2, 1.5 μM IF3, 0.5 μM Bpy-Met-tRNA^fMet^ (Bpy-tRNAi), 0.2 mM guanosine nucleotides, and varying concentrations of the tested ligand. Fluorophore excitation and measurement of the emitted fluorescence, together with IR-mediated perturbation of the equilibrium, are achieved through an objective and dichroic mirror. (C) Time courses of fluorescence showing the equilibrium relaxation upon thermal perturbation at varying ligand concentrations. Blue vertical lines indicate the fluorescence signal readout prior to the equilibrium perturbation (F_i_), while vertical red lines indicate the fluorescence signal upon reaching the new equilibrium (F_f_). Colored traces represent different concentrations of the titrating mRNA (0.1 to 4 μM). The ratio between the F_f_ over F_i_ times 1,000 is the normalized thermophoresis shift (MST). The dependency of MST shift on ligand concentration allows to estimate *K*_*D*_ constants of the interaction using a hyperbolic or quadratic function and nonlinear regression fitting. MST measurements (closed circles) are indicated in the same colors as their respective time traces in (C). (D) Formation of 30S IC measured by MST. Time traces of thermophoresis (S1 Fig) were used to calculate MST values for all four combinations of mRNA start codons and labeled tRNAs for dependencies on mRNA concentration. Squares indicate 30S ICs programmed with AUG mRNA, while circles show that UUC was used as start site. Closed symbols indicate that Bpy-Met-tRNA^fMet^ was used, while open symbols correspond to Bpy-Phe-tRNA^Phe^. Continuous lines indicate nonlinear regression fittings. Three to four measurements were performed; mean and error bars representing standard deviations are plotted (S1 Data). Bpy, BODIPY fluorophore; DksA, RNA polymerase-binding transcription factor DksA; GDP, guanosine diphosphate; GTP, guanosine triphosphate; IC, initiation complex; IF1, translation initiation factor IF1; IF2, translation initiation factor IF2; IF3, translation initiation factor IF3; IR, infrared; MST, Microscale Thermophoresis; (p)ppGpp, guanosine tetra- and pentaphosphate; RelA, (p)ppGpp synthase RelA; SpoT, Bifunctional (p)ppGpp synthase/hydrolase SpoT; tRNAi, initiator tRNA.

During colonization, pathogenic bacteria reshape their transcriptome and proteome to activate virulence genes, promote tissue-associated biofilm, and enter dormancy, ultimately increasing aggressiveness, antibiotic tolerance, and persistence of the pathogen (reviewed in [1,2]). Host colonization entails fluctuations in nutrient availability that generally trigger stringent response in bacteria (Fig 1A). Stringent response is mediated by the ribosome-associated RelA/SpoT homolog protein superfamily and leads to the accumulation of the hyperphosphorylated guanosine nucleotides, collectively called (p)ppGpp (Fig 1A) [3]. Impaired (p)ppGpp production results in antibiotic sensitivity, low biofilm formation, and low pathogenicity of several bacteria, making the stringent response an appealing target for antibiotic development [4–6]. mRNA translation to proteins requires the action of several guanosine nucleotide-binding factors, on and off the ribosome. Although (p)ppGpp have been shown to bind translational GTPases [7–12], the extent of inhibition and subsequent effect on protein synthesis regulation remain controversial.

The initiating ribosome orchestrates a complex equilibrium involving three initiation factors (IFs), mRNA, and initiator tRNA (fMet-tRNA^fMet^, from here in tRNAi) to maximize the speed and accuracy of start codon selection, ultimately defining the reading frame for mRNA translation [13]. The translational guanosine triphosphate (GTP)-binding factor IF2 recruits tRNAi to the 30S pre-initiation complex (pre-IC), accompanies the subsequent isomerization to the 30S IC (start codon recognition), promotes the association of the large 50S subunit, and occupies all intermediates of the 70S pre-IC leading to a ready-to-elongate 70S IC [13–16]. Although GTP has been shown to enhance IF2 activity, hydrolysis of GTP appears to be dispensable [17]. IF2 shows a broad spectrum of binding properties as a function of the bound guanosine nucleotide, enabling cycling between high- and low-affinity states: GTP contributes to 30S IC assembly, whereas guanosine diphosphate (GDP) allows dissociation of the factor from the 70S pre-IC [14,16,18]. Both the dispensability of GTP hydrolysis and the wide range of affinities displayed by the factor indicate that IF2 may function as a molecular sensor of guanosine nucleotides. Indeed, IF2 was shown to be preferentially affected by ppGpp among translational GTPases, inhibiting translation initiation and the first peptide bond formation [9]. Whether ppGpp stringently or permissively arrests translation initiation or whether IF2 is able to use pppGpp remained open questions. (p)ppGpp-dependent activation of genes and involvement in controlling the growth rate of the cell by acting on DNA, RNA, and protein synthesis [19–22] argue for a permissive mechanism. Here, we use advanced fluorescence spectroscopy techniques to investigate how the initiating ribosome copes with (p)ppGpp accumulation to translate mRNAs, ultimately allowing cell survival by reshaping the bacterial proteome during environmental adaptation.

## Results

### Experimental approach

To monitor the interaction of initiator tRNA and 30S ribosomal subunits, we used Microscale Thermophoresis (MST) with a fluorescently labelled initiator tRNA (Bpy-tRNAi) (Fig 1B). MST allows to measure fluorescence in a constrained space. Changes on the readout derive from molecular drifts resulting from small thermal equilibrium perturbations [23]. Upon equilibrium perturbations, the patterns of Bpy-tRNAi migration away from the observation space are related to bound and unbound states (Fig 1B) [23,24]. Binding of Bpy-tRNAi to 30S complexes resulted in an increase of thermophoresis as compared to the free Bpy-tRNAi (Fig 1C). On the contrary, a decrease in thermophoresis indicated the dissociation of Bpy-tRNAi. The relation between fluorescence measured at both equilibriums (F_f_ to F_i_, after and prior to the perturbation, respectively) as a function of ligand concentration allowed to determine dissociation constants of the interaction (Fig 1C) [23,24].

By observing the bound state of Bpy-tRNAi to the 30S subunit, MST could be measuring either IC, 30S pre-IC, or 30S IC, as they are identical in terms of ligand composition [25]. Decoding of the start codon leads to formation of the 30S IC and was shown to increase the kinetic stability of tRNAi [25]. To unambiguously assign which step is monitored by MST, we used different combinations of start codons and fluorescent tRNAs. Titrations with an mRNA bearing a canonical AUG resulted in increased MST (Fig 1D). On the contrary, mRNAs with a UUC codon at the start site showed negligible thermophoresis signal when Bpy-tRNAi was used, indicating that MST monitors the formation of the 30S IC rather than the 30S pre-IC. To further validate this observation, we used a Bpy-Phe-tRNA^Phe^ with the corresponding UUC codon at the start site, which resulted in an increase of thermophoresis, consistent with IF2 requiring an N-blocked aminoacyl tRNA for translation initiation [26,27]. Accordingly, if an AUG codon was used at the start site, negligible MST amplitude was observed (Fig 1D). Altogether, MST allows to monitor the formation of 30S complexes that have decoded the start site, 30S IC, rather than previous steps.

### Guanosine nucleotides as cofactors of IF2

**Fig 2:**
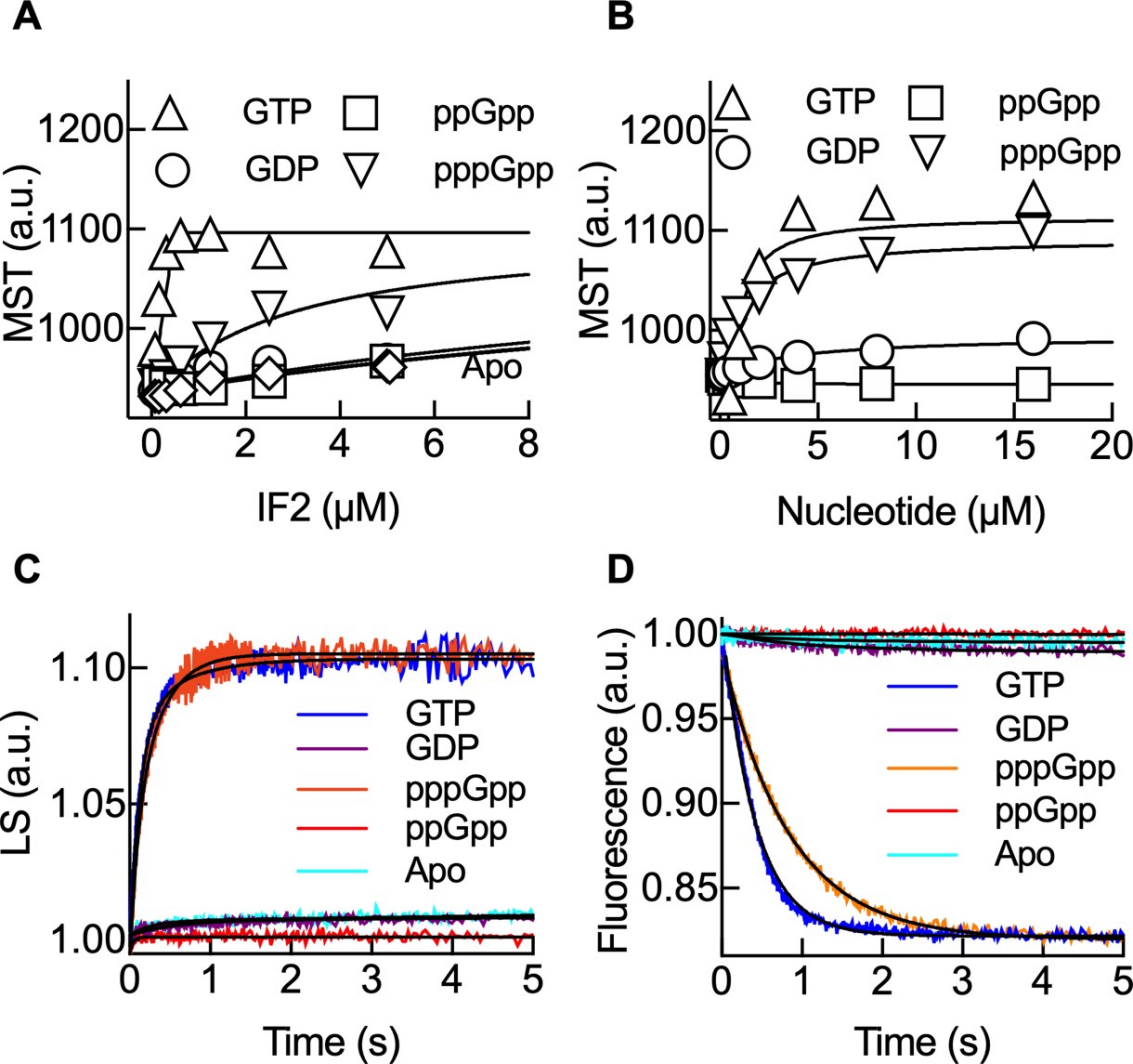
pppGpp allows translation initiation. (A) Formation of 30S IC measured by MST at increasing concentrations of IF2 in the presence of ppGpp (squares), pppGpp (down triangles), GTP (up triangles), GDP (circles), or in the absence (diamonds) of guanosine nucleotides. Continuous lines indicate nonlinear regression fittings with a quadratic equation. Three measurements were performed; mean and standard deviation are plotted (S1 Data). (B) as (A) to measure the concentration dependence for each tested guanosine nucleotide at constant IF2 (1 μM). (C) Formation of 70S pre-IC as measured by light scattering (LS) on a stopped-flow apparatus [29]; 0.1 μM 30S ICs were formed in the absence of (cyan) or the presence of 0.2 mM ppGpp (red), pppGpp (orange), GTP (blue), or GDP (purple) and rapidly mixed with 0.3 μM 50S subunits. Continuous lines show best fits using exponential functions. (D) Formation of 70S IC as measured by Bpy-tRNAi accommodation in the P site of the ribosome; 0.1 μM 30S ICs were formed with Bpy-tRNAi and rapidly mixed with 0.3 μM 50S subunits on a stopped-flow apparatus as described in [14]. Colors are as in (C). Continuous lines show best fits using an exponential function for a single reaction step. Each stopped-flow trace results from the mean of 5 to 7 replicates. Bpy-tRNAi, Bodipy labelled initiator tRNA; GDP, guanosine diphosphate; GTP, guanosine triphosphate; IC, initiation complex; IF2, translation initiation factor IF2; MST, Microscale Thermophoresis.

IF2 was shown to bind GDP and GTP with affinities in the μM range, while ppGpp competed with GTP efficiently in a similar range of affinities [8,9,28]. IF2 binds the 30S through two interactions: the first, rather unspecific, is driven by the N domain, while the second is GTP- and IF1-dependent and occurs through the G domain [16]. Binding of GDP and GTP to the 30S-bound IF2 confer different conformations to the factor, ultimately influencing 50S association if IF3 is absent [18]. Here, we first measured 30S IC formation as a function of IF2 concentration in the presence of different guanosine nucleotides. GTP usage increased the amplitude of thermophoresis stoichiometrically with IF2, while pppGpp required higher IF2 concentration to reach the thermophoresis amplitudes observed in the GTP case (Fig 2A). In the presence of GDP, ppGpp or without any nucleotide (Apo), the amplitude of thermophoresis was lower (Fig 2A). Our results indicate that IF2 can use GTP or pppGpp for the recruitment of tRNAi and promote 30S IC formation, albeit with a higher factor requirement for the alarmone. EC_50_ binding concentrations for Bpy-tRNAi were high (>1 μM) for IF2 bound to ppGpp, GDP, or in the absence of any guanosine nucleotide (Apo), similar to measurements of IF2-Bpy-tRNAi-GNP ternary complex formation in the absence of the 30S subunit (S2 Fig). Thus, ppGpp and GDP may program IF2 to destabilize tRNAi on the 30S complex, precluding 30S IC formation. Consistently, guanosine nucleotide titrations showed thermophoresis increase for GTP and pppGpp, whereas GDP and ppGpp failed to promote Bpy-tRNAi binding at any concentration (Fig 2B). The calculated affinities of GTP and pppGpp for the IF2-bound 30S IC were similar (*K*_*D*_ = 0.5 ± 0.1 μM). GTP and pppGpp seem to activate IF2 to promote 30S IC formation, whereas GDP and ppGpp prevent IF2 from recruiting the initiator tRNA and/or to transit towards 30S ICs. Notably, pppGpp can program IF2 to promote 30S IC formation, albeit at higher factor concentrations, indicating the alarmone may confer yet another conformation to IF2.

To investigate the transition to translation elongation as a function of IF2-bound nucleotides, we monitored the formation of the 70S pre-IC by light scattering (LS) and 70S IC formation by the fluorescence of Bpy-tRNAi accommodation using stopped-flow techniques [14,29] (Fig 2C and 2D). Mixing of 50S subunits with 30S complexes formed with GTP or pppGpp resulted in a rapid increase of LS over time (*k*_app_ = 2.9 ± 0.3 s^–1^), indicating that 70S pre-ICs are readily formed (Fig 2C). The subsequent 70S IC complex, as measured by Bpy-tRNAi accommodation, showed a decrease in fluorescence over time following 50S subunit joining (*k*_app_ = 2.4 ± 0.2 s^–1^) (Fig 2D) for GTP. When pppGpp was used, Bpy-tRNAi accommodated 2-fold slower (*k*_app_ = 1.2 ± 0.2 s^–1^). Altogether, GTP programs IF2 to form a 30S IC that is capable of rapidly binding the 50S subunit and accommodating tRNAi in the 70S IC to accept the incoming elongator aminoacyl tRNAs. pppGpp-bound IF2 is able to promote 70S pre-IC and 70S IC formation at a kinetic cost. The omission of nucleotides or presence of GDP or ppGpp showed reduced efficiencies (amplitude) for both reactions (Fig 2C and 2D). Although GDP and ppGpp could compete with GTP for IF2, cellular concentrations of GDP are low at any cell growth condition [30,31], whereas (p)ppGpp accumulate to millimolar concentrations during bacterial stringent response [30,32]. Overall, guanosine nucleotides act as cofactors of IF2, modulating its capacity to position the initiator tRNA along the pathway of translation initiation (GTP/GDP) or as messengers of stringent response (GTP/pppGpp/ppGpp).

### mRNA dependence of ppGpp inhibition

**Fig 3:**
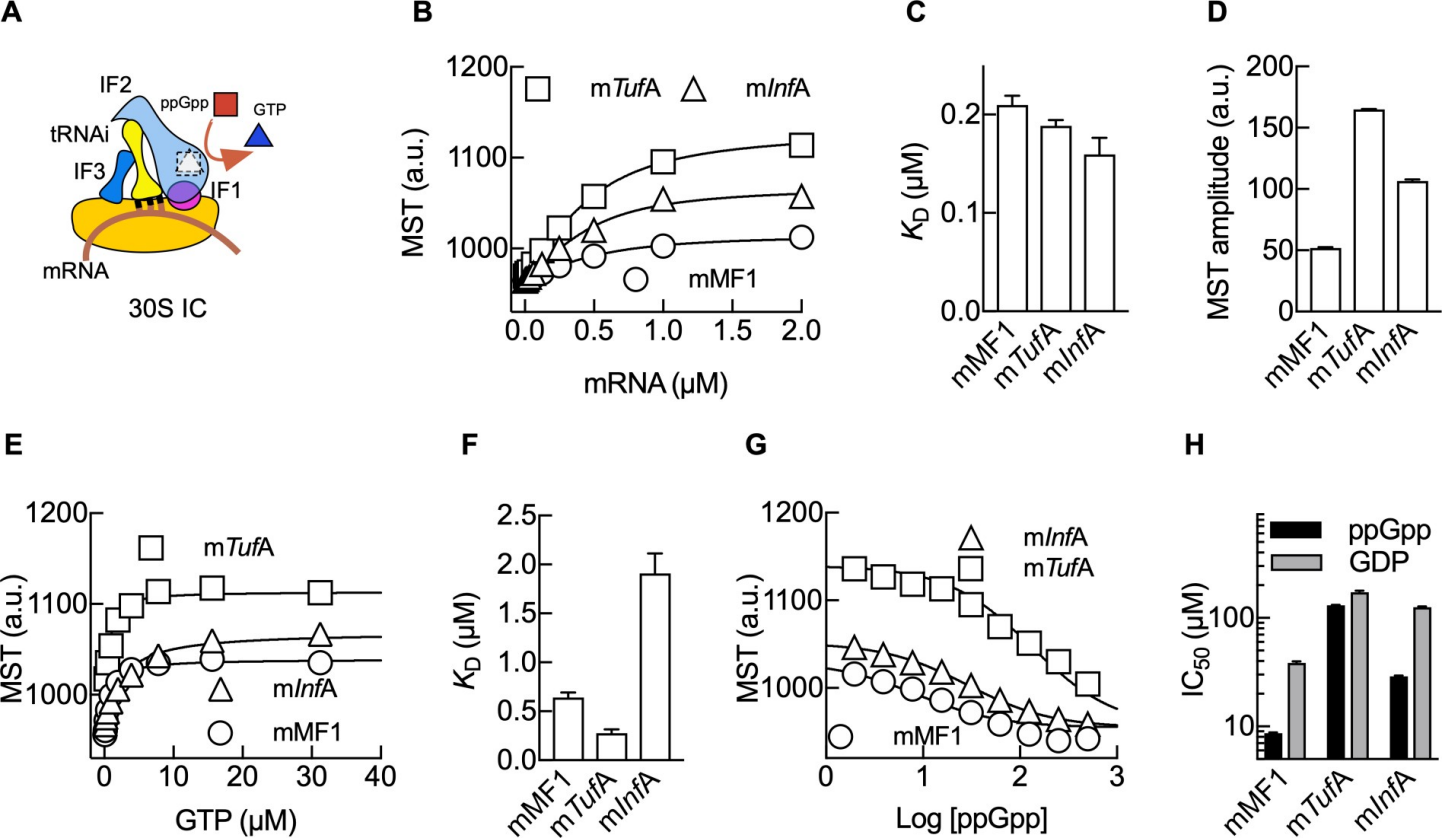
MST analysis shows an mRNA dependence of 30S IC formation. (A) Scheme of the 30S IC highlighting ppGpp (red square) and GTP (blue triangle) competition for IF2. (B) MST dependence on the mRNA concentration for m*Tuf*A (squares), m*Inf*A (triangles), or model messenger mMF1 (circles). MST was calculated from the respective fluorescence time dependencies (S3 Fig). Comparison of the resulting *K*_*D*_ (C) and MST amplitudes (D) as an indicator of the efficiency of 30S IC formation for all three mRNAs. (E) Formation of 30S IC with increasing concentrations of GTP for the three mRNAs. Symbols as in (B). (F) Comparison of *K*_D_ values calculated from (E). (G) ppGpp to GTP competitive assays for 30S IC formation. The 30S ICs formed with each mRNA (symbols as in B) in the presence of 50 μM GTP were subjected to increasing concentrations of ppGpp. Log of competitor concentrations is plotted and used for determining the inhibitory concentration for 50% inhibition (IC_50_) using a same-site competition model. (H) Bar graph comparing IC_50_ values for ppGpp (black) or GDP (gray) for all three mRNAs (S4 Fig). Continuous lines indicate nonlinear regression fittings with quadratic (B and E) or same-site competition (G) functions. Three measurements were performed; mean and standard deviations are plotted (S1 Data). GDP, guanosine diphosphate; GTP, guanosine triphosphate; IC, initiation complex; IF1, translation initiation factor IF1; m*Inf*A, *Inf*A mRNA; MST, Microscale Thermophoresis; m*Tuf*A, *Tuf*A mRNA; tRNAi, initiator tRNA.

ppGpp accumulation during bacterial starvation was shown to inhibit IF2 and protein synthesis; however, a number of genes are activated, and their mRNAs are translated in vivo at high ppGpp concentrations [9,33]. A model bridging these sets of observations was lacking. mRNAs may contain the determinants to allow translation initiation at otherwise inhibiting concentrations of ppGpp, allowing GTP to compete with ppGpp (Fig 3A). To test this model, we used two housekeeping mRNAs, m*Tuf*A and m*Inf*A, coding for the essential proteins elongation factor EF-Tu and initiation factor IF1, respectively. The former is 40-fold more abundant than the latter and appears 2–3-fold up-regulated at the translational level in *Escherichia coli* [33,34]. Additionally, we included a derivative of the 022 model mRNA (mMF1) with a shorter coding region that was used in previous studies of ppGpp and translation initiation studies [9,35]. 30S IC formation as a function of mRNA concentration was increased for m*Tuf*A in comparison to m*Inf*A or the model mMF1, as evaluated by the thermophoresis amplitudes (Fig 3B). At the same time, analysis of the concentration dependence resulted in similar affinities for all mRNAs. This is consistent with a model in which mRNAs bind similarly to the 30S pre-IC; however, they promote 30S IC formation with different efficiencies (Fig 3C and 3D). In agreement, GTP titrations in complexes formed with each mRNA showed the same thermophoresis trend for 30S IC formation: m*Tuf*A *>* m*Inf*A *>* mMF1 (Fig 3E). Additionally, equilibrium dissociation constants for GTP differed around an order of magnitude as a function of the mRNA used. The calculated *K*_*D*_ of GTP for the 30S IC programmed with m*Tuf*A was about 7-fold lower (*K*_D_ = 0.28 ± 0.04 μM) than that for m*Inf*A (*K*_D_ = 1.9 ± 0.2 μM) and about 2-fold lower than that for mMF1 (*K*_D_ = 0.65 ± 0.05 μM) (Fig 3F). Thus, m*Tuf*A, which codes for the highly abundant EF-Tu, possesses functional determinants that allow efficient 30S IC formation with IF2 binding GTP with higher affinity, whereas m*Inf*A results in less efficient 30S IC formation and weaker binding of GTP.

ppGpp competition experiments with GTP showed a decrease in 30S IC formation for all mRNAs with increasing concentrations of ppGpp, however, with different dependencies on the competing nucleotide (Fig 3G). The calculated inhibitory concentrations (IC_50_) ranged over 15-fold, with m*Tuf*A being the least sensitive to ppGpp (IC_50_ = 132 ± 1 μM), mMF1 the most sensitive (IC_50_ = 8.6 ± 0.2 μM), and m*Inf*A being inhibited with an intermediate concentration (IC_50_ = 29 ± 1 μM) (Fig 3H). Similar experiments performed with GDP required higher concentrations of the competitor to inhibit 30S IC; however, the mRNA dependence was maintained (Fig 3H and S4 Fig). Thus, ppGpp-mediated inhibition of translation initiation is dynamic and dependent on the mRNA bound to the 30S complex, with the highly translated m*Tuf*A being more tolerant to ppGpp than m*Inf*A.

### ppGpp-mediated regulation of 70S IC progression

**Fig 4:**
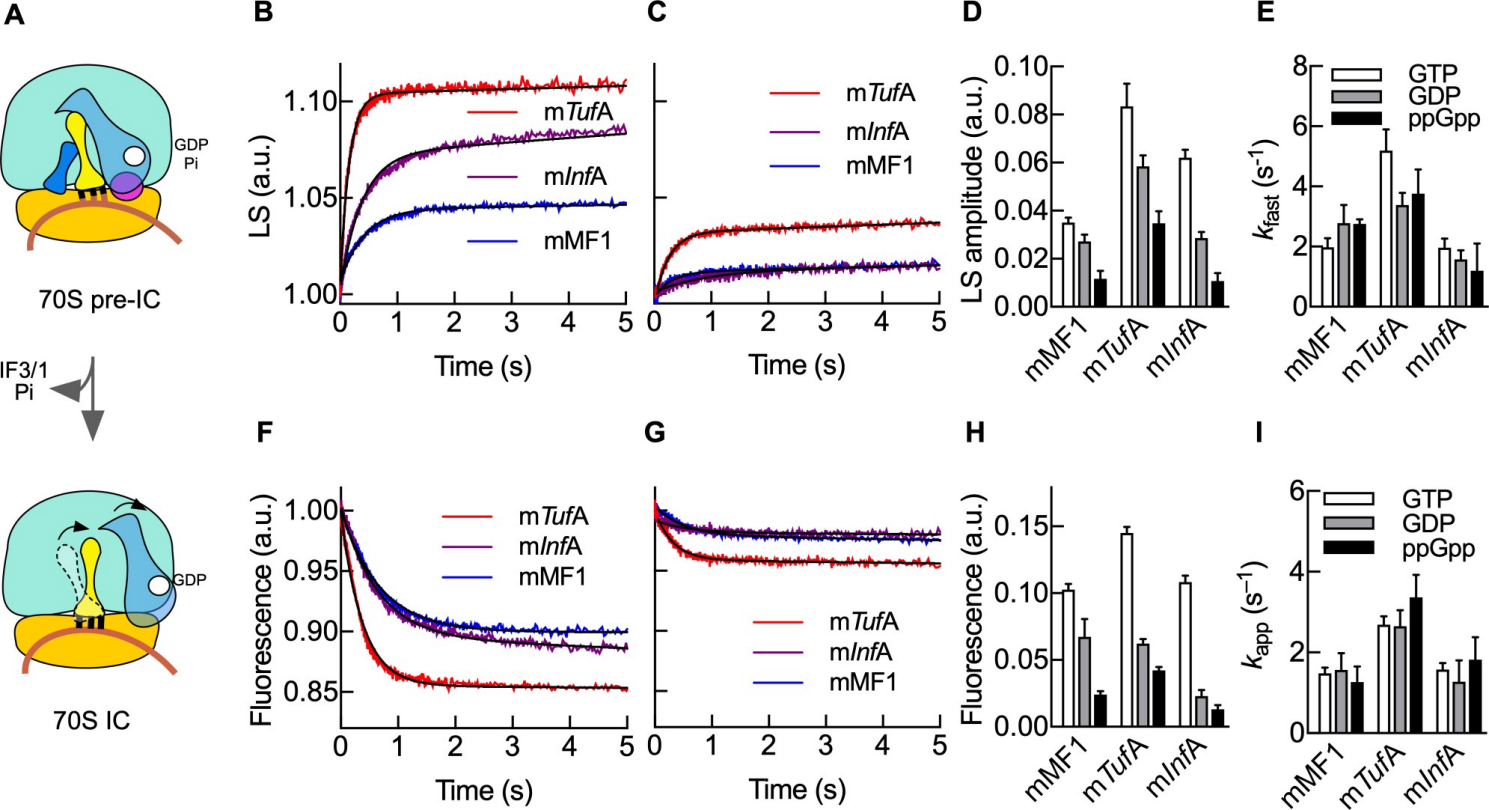
Kinetic parameters of ppGpp-mediated regulation of 70S IC progression. (A) Scheme of 70S IC formation. The 30S complexes programmed with m*Tuf*A (red), m*Inf*A (purple), or mMF1 (blue) and 20 μM GTP in the absence (B) or presence (C) of 200 μM ppGpp were mixed with 50S subunits. Time traces were analyzed by nonlinear regression with exponential terms. (D) Bar graph comparing amplitudes in the absence of any competing nucleotide (white) or in the presence of ppGpp (black) or GDP (gray). (E) Bar graph comparing apparent rates of 70S pre-IC formation (colors as in D). Formation of 70S IC, as measured by Bpy-tRNAi accommodation in the absence (F) or presence of 200 μM ppGpp (G). Time traces were analyzed by nonlinear regression with one exponential term. (H) Bar graph comparing amplitude variations as a function of mRNAs and guanosine nucleotides (colors as in D). (I) Bar graph comparing apparent rates of 70S IC formation for all three mRNAs (bar colors as in D). Continuous lines show best fits using an exponential function for a single reaction step. All time traces are mean values of 5 to 10 replicates. Bar graphs show mean and standard errors (error bars) derived from the nonlinear regression fitting (D,E,H,I) (S1 Data). Bpy-tRNAi, Bodipy labelled initiator tRNA; GDP, guanosine diphosphate; GTP, guanosine triphosphate; IC, initiation complex; IF3, translation initiation factor IF3; LS, light scattering; m*Inf*A, *Inf*A mRNA; m*Tuf*A, *Tuf*A mRNA.

Late events of translation initiation entail the association of the 30S IC with the large ribosomal subunit 50S, leading to the intermediate 70S pre-IC, which after IFs dissociate results in a ready-to-elongate 70S IC (Fig 4A) [14]. Here, we measured the velocities of 70S pre-IC formation as a function of the bound mRNA, the IF2-bound nucleotide, and the ability of ppGpp to compete with GTP (Fig 4). Rapid kinetics analysis of 50S joining to 30S ICs shows that complexes programmed with m*Tuf*A resulted in higher 70S pre-IC formation than those formed with m*Inf*A or mMF1, essentially following the same trend as observed by thermophoresis analysis (Fig 4B). Addition of ppGpp as a competitor for GTP showed an overall decrease of 70S pre-IC formation efficiency for all mRNAs; however, complexes harboring m*Tuf*A appeared to be the least affected while m*Inf*A were the most affected (Fig 4C). Using GDP as a competitor for GTP also resulted in a decreased efficiency of 70S pre-IC formation, albeit inhibiting the reaction to a lesser extent than ppGpp (Fig 4D and S5 Fig). Additionally, the formation of the 70S pre-IC appears to be kinetically influenced by m*Inf*A, showing 2- to 3-fold slower velocities than m*Tuf*A or mMF1 (Fig 4E). However, the nucleotide competing with GTP, either GDP or ppGpp, did not perturb the initial rate of the 50S joining to 30S ICs, suggesting that the observed fraction corresponds to 30S ICs containing GTP (Fig 4E). Hence, ppGpp competition with GTP results in fewer 30S ICs habilitated to recruit the 50S subunit; albeit, mRNAs modulate the GTP-bound fraction. Similar reactions in the absence of GTP resulted in negligible rapid 70S pre-IC formation for ppGpp and some transitions for GDP (S6 Fig).

IF2 populates every intermediate of the multistep reaction leading to 70S IC formation, ultimately promoting the accommodation of tRNAi in the P site before factor dissociation (Fig 4A) [14,36]. Formation of 70S IC was assessed by measuring Bpy-tRNAi accommodation using the stopped-flow technique as a function of the guanosine nucleotide competing with GTP for all three mRNAs (Fig 4). In the absence of any GTP competitor, tRNAi accommodated rapidly after 50S association (Fig 4F), with overall efficiencies reflecting those of 30S IC formation, as measured by thermophoresis (Fig 3B), or 70S pre-IC, as measured by LS (Fig 4B). Replacement of GTP by GDP or ppGpp resulted in small fluorescence changes for tRNA accommodation, indicating that very few mMF1 or m*Inf*A coded 30S complexes contained the tRNAi (S7 Fig). However, when m*Tuf*A was used, a reasonable fraction of complexes was allowed to accommodate the tRNAi towards 70S ICs (S7 Fig). Addition of GDP or ppGpp as competitors of GTP resulted in a defined decrease of overall efficiencies of tRNAi accommodation; however, ppGpp showed a higher degree of inhibition than GDP (Fig 4G and S5 Fig). As observed for 70S pre-IC formation, complexes programmed with m*Tuf*A appeared to be less sensitive to ppGpp, while those programmed with m*Inf*A showed more susceptibility (Fig 4H). Nonlinear analysis of the time dependencies shows that the tRNAi accommodates at different rates for each mRNA. Although the extent of the reaction is affected by the competing nucleotide, the apparent rates of tRNAi accommodation appeared unaffected, indicating that the resulting amplitude corresponds to GTP-bound 30S ICs (Fig 4I). Altogether, the formation of 70S ICs are halted by ppGpp acting at a prior step, 30S IC formation. However, the extent of the progression towards protein synthesis is mediated by the competition between GTP with ppGpp in an mRNA-dependent manner.

### Permissive mRNA translation with ppGpp

**Fig 5:**
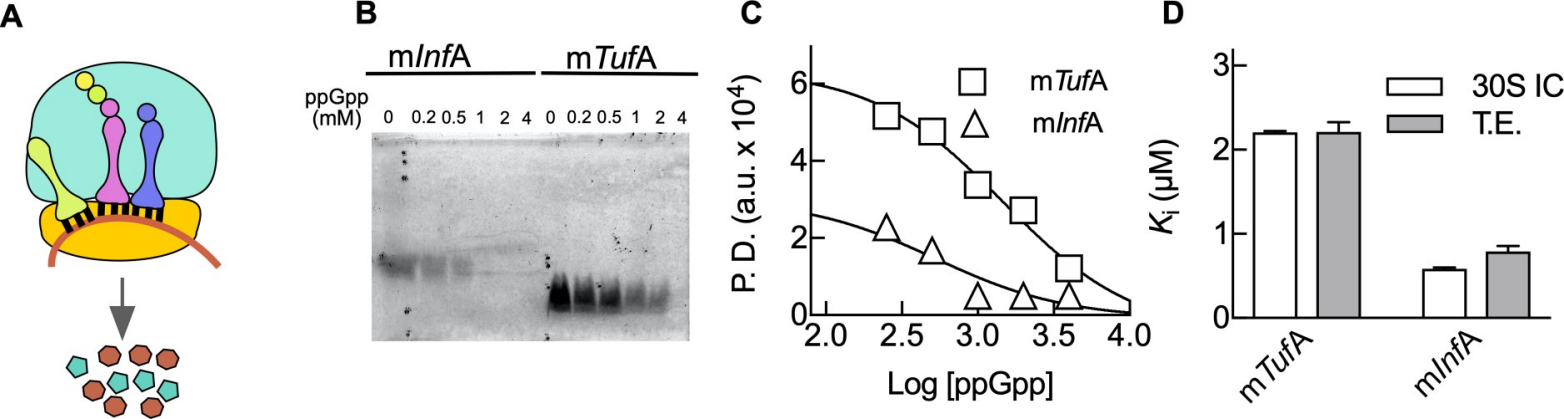
Permissive ppGpp-mediated inhibition of mRNA translation. (A) Scheme of the translating 70S complex. (B) In vitro translation of m*Tuf*A and m*Inf*A derivatives harboring a coding sequence for the Lumio labelling system (Experimental conditions and analysis). Protein synthesis reactions were started in the presence of 2 mM GTP and increasing concentrations of ppGpp. Synthetized proteins were fluorescently labelled and resolved by 20% SDS-PAGE. The resulting images were analyzed by pixel densitometry using ImageJ [37] to estimate translation efficiencies (TEs). (C) Log of ppGpp concentrations was plotted and used for determining the inhibitory concentration for 50% inhibition (IC_50_) using a same-site competition model. (D) Comparison of inhibitory constants for both mRNAs as measured during 30S IC formation (white) or overall translation efficiency (gray). Mean and standard deviations from three measurements are plotted (S1 Data). GTP, guanosine triphosphate; m*Inf*A, *Inf*A mRNA; m*Tuf*A, *Tuf*A mRNA; TE, translation efficiency.

Our results are consistent with IF2 sensing ppGpp to GTP ratios in an mRNA-dependent manner and ultimately allowing translation at varying protein output efficiencies (Fig 5A). To test this premise, we used a pure cell-free translation system at physiological concentrations of ribosomes, aminoacyl-tRNAs, translational GTPases, GTP, and varying ppGpp concentrations (up to 4 mM) (Fig 5B). In the absence of ppGpp, translation of m*Tuf*A was 3-fold higher than m*Inf*A, consistent with our results obtained by measuring every previous step, 30S IC, 70S pre-IC, and 70S IC formation (Figs 3 and 4). Addition of ppGpp resulted in decreased translation efficiencies for both mRNAs in a ppGpp concentration-dependent manner (Fig 5C). The inhibitory concentration IC_50_ differed for each mRNA, with m*Tuf*A being 4-fold more tolerant to ppGpp than m*Inf*A (Fig 5D).

The calculated affinity of GTP (Fig 3) for complexes differing on the programmed mRNA allowed to estimate the ppGpp inhibitory constants for both 30S IC formation and translation efficiencies. Both inhibitory constants were similar, supporting a model in which IF2 is a primary target for ppGpp-mediated regulation during protein synthesis (Fig 5D) [9]. Remarkably, m*Tuf*A was translated at ppGpp concentrations that have been reported during cell starvation [30,31], indicating that the protein synthesis apparatus is capable of tolerating high concentrations of the alarmone. Altogether, the protein synthesis apparatus can translate mRNAs at physiological concentrations of ppGpp; however, the initiating ribosome is capable of sorting which mRNAs shall enter the elongation phase of protein synthesis.

### Structural determinants for mRNA translation at high ppGpp

**Fig 6:**
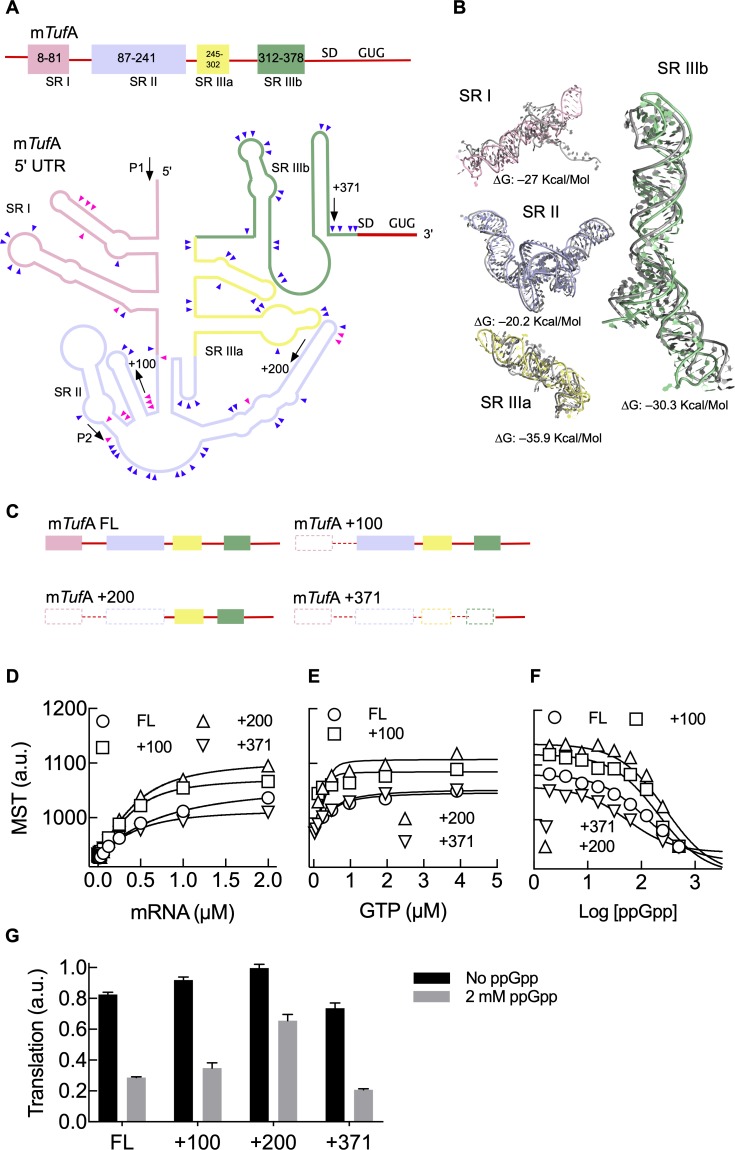
m*Tuf*A structured enhancer of translation initiation. (A) Two-dimensional representation of the m*Tuf*A 5′ UTR. The RNAfold algorithm predicted three structured regions: SR I (pink), SR II (violet), SR IIIa (yellow), and SR IIIb (green). Blue and pink arrows indicate adenines or cytidines found to be unpaired in vivo by DMS chemical probing [38]. Blue arrows indicate a match between modelled structures and in vivo chemical probing, whereas pink arrows indicate a lacking match. Black arrows indicate the starting points of the mRNA truncations used here and in vivo active promoters. (B) Three-dimensional modelling of the three structured elements contained in the m*Tuf*A 5′ UTR. Three-dimensional modelling was performed either using the full-length (FL) UTR (gray) or the isolated structured regions (colored as in (A)). (C) Scheme representing the different m*Tuf*A truncations used in this study. FL stands for the UTR used in Figs 3–5. Translation initiation was measured by MST as a function of the mRNA (D) or GTP (E) for all m*Tuf*A truncations. (F) ppGpp to GTP competition experiments as measured by MST. (G) In vitro translation efficiencies of m*Tuf*A derivatives in the absence or presence of 2 mM ppGpp. Mean and standard deviations from three measurements are plotted in D, E, F, and G (S1 Data). DMS, dimethyl sulfide; GTP, guanosine triphosphate; MST, Microscale Thermophoresis; m*Tuf*A, *Tuf*A mRNA; SR, structured region.

m*Tuf*A, in contrast to m*Inf*A, promoted higher yields of 30S IC formation, induced IF2 to be more tolerant to ppGpp, and could be translated at physiological concentrations of the alarmone (see above). In the cell, m*Tuf*A is produced by three different promoters. The *Str* promoter allows the transcription of the entire *Str* operon where *Tuf*A is the last coded gene. On the other hand, two promoters upstream of the *Tuf*A initiation codon contain a long untranslated region (UTR), 397 nucleotides (nt) long for the first (P1) and 254 nt for the second (P2) promoter. Here, we use bioinformatic and biochemical analysis of the 30S-bound 5′ UTR of m*Tuf*A to inquire on the mechanism leading to mRNA translation under stringent response conditions (Fig 6).

**Fig 7:**
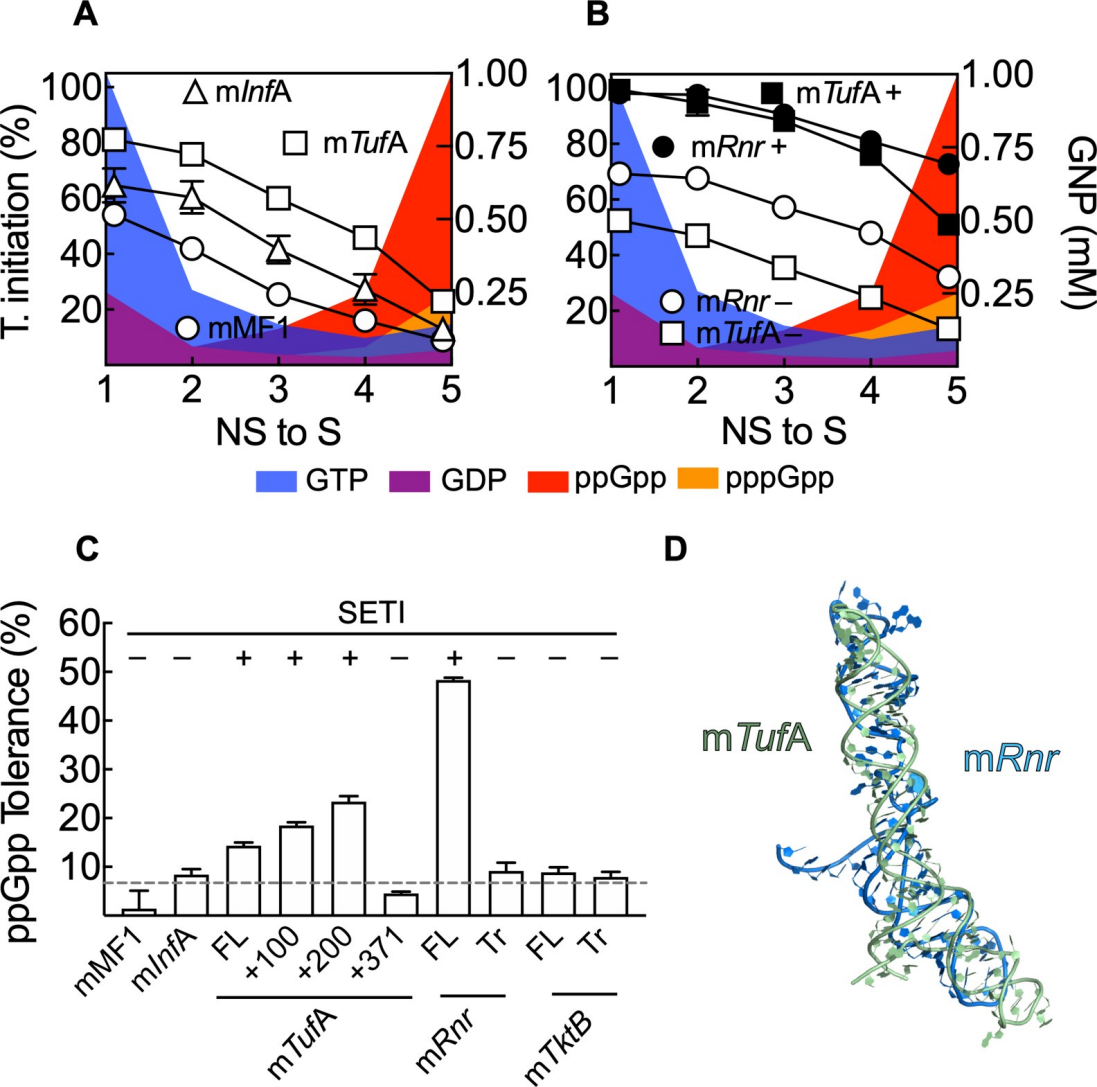
SETIs and ppGpp tolerance. (A) Translation initiation efficiency as a function of mRNAs along five steps of transition from NS to S conditions. Nucleotide concentrations are indicated by the filled areas and refer to the right axis, GTP (blue), GDP (violet), ppGpp (red), and pppGpp (orange). m*Tuf*A (square), m*Inf*A (triangles), and mMF1 (circles) represent averages of three replicates (S1 Data). (B) as in (A) for m*Tuf*A (squares) or m*Rnr* (circles) variants containing (close) or lacking (open) the SETI element proximal to the TIR. Symbols and error bars represent averages of three replicates and standard deviations, respectively (S1 Data). (C) Comparison of ppGpp tolerance as calculated from the ratio of translation initiation efficiencies found in the presence of 0.2 mM ppGpp over the efficiencies obtained in the absence of the alarmone (S1 Data). Translation initiation efficiencies for all mRNAs tested here were calculated from thermophoresis measurements considering the highest observed MST (+200 m*Tuf*A construct) as 100% and that of free Bpy-tRNAi as 0%. All 30S IC components are as in Fig 1. Dashed line indicates the average tolerance of all mRNAs lacking the SETI element. FL stands for full-length while Tr indicates a truncation of the SETI element in m*Rnr* or the corresponding segment in m*Tkt*B. (D) Overall folding comparison of the m*Tuf*A and m*Rnr* SETIs. Both mRNA segments share similar folds, albeit having a primary sequence identity below 30% (see S2 File for primary and secondary structure comparisons). Bpy-tRNAi, Bodipy labelled initiator tRNA; GDP, guanosine diphosphate; GNP, guanosine nucleotide; GTP, guanosine triphosphate; IC, initiation complex; m*Inf*A, *Inf*A mRNA; m*Rnr*, *Rnr* mRNA; MST, Microscale Thermophoresis; m*Tkt*B, *Tkt*B mRNA; m*Tuf*A, *Tuf*A mRNA; NS, non-stringent; S, stringent; SETI, structured enhancer of translation initiation; TIR, translation initiation region.

The translation initiation region (TIR) of m*Tuf*A is composed by a rather weak Shine-Dalgarno (SD) sequence in addition to the less frequently used GUG initiation codon. Secondary structure predictions indicated that m*Tuf*A UTR folds into three structured regions (SR I–III, Fig 6A). In addition, SR III showed two separated segments, (a) and (b), with the latter folding into two particularly consistent hairpins (Fig 6A and S1 File). Structural predictions of the isolated SRs showed similar secondary structures as compared to modelling the full-length (FL) UTR (Fig 6A and S1 File). Free energy calculations at the experimental conditions yielded ΔG values ranging from −20 to −36 kcal/mol for the isolated SRs, indicating that the acquired structures are relatively stable (S1 File). Comparisons between two different prediction algorithms, RNAfold and mFold, showed high consistency for SR IIIb and to a lesser extent for SR I and SR IIIa. SR II differed between mFold and RNAfold (S1 File). To verify if the predicted structures are also represented in the cell, we used the in vivo dimethyl sulfide (DMS) probing data sets from the C. Gross group [38]. DMS probing allows to identify unpaired adenines or cytidines in a given RNA structure. From a total of 85 in vivo unpaired nucleotide signals, 63 were found to match our structural prediction, while the 22 that did not clustered in SR I and SR II (Fig 7A). This may be explained as in vivo these regions are part of the preceding coding sequence in the *Str* polycistronic mRNA. Thus, the lack of correlation between modeling SR I and SR II and in vivo data may result from the translation of the preceding coding region with ribosomes opening the mRNA structures. On the other hand, all DMS signals matched our structural prediction of SR III, indicating that all three cellular transcripts of m*Tuf*A fold the SR III in vivo. Structural modelling incorporating in vivo probing showed identical folds for SR III (S1 File) [38]. Two-dimensional predictions from RNAFold were used to model the tertiary structure, showing high consistency between the fully modelled UTR and the isolated SRs (Fig 6B). Root mean square deviation (RMSD) values for these comparisons ranged from 4 to 20 Å, relatively low if taking into consideration the mass of the molecules.

To study how the SRs of m*Tuf*A influence 30S IC formation and mRNA translation, we constructed truncations aiming to disrupt SR I (+100), SR I and SR II (+200), and all three (+371) SRs. However, all m*Tuf*A variants maintained the TIR (SD, spacer, and start codon) and coding sequence (Fig 6C). Formation of 30S IC was measured as a function of the mRNA, GTP, and competing ppGpp by thermophoresis (Fig 6D–6F). All three m*Tuf*A constructs showed similar affinities for the 30S (*K*_*D*_ = 0.25 ± 0.05), albeit resulting in different extents of 30S IC formation (Fig 6D). Removal of the first 100 and 200 bases resulted in greater 30S IC formation if compared to the FL construct. Removal of all three SRs diminished 30S IC formation. Thus, SR III appears to enhance 30S IC formation while SR I counterbalances such enhancement in vitro. Interestingly, the P2 promoter of the *Tuf*A gene transcribes an mRNA lacking SR I. Our data could indicate that, in vivo, this m*Tuf*A variant may outperform others in terms of translation efficiency. In addition, disruption of SR I and II resulted in increased affinities for GTP, while removal of all SRs resulted in decreased enhancement of 30S IC formation as a function of GTP (Fig 6E). mRNAs lacking SR I and II promoted a 2- and 3-fold increase of ppGpp tolerance, respectively (IC_50_ = 200 to 300 μM). Removal of SRs I–III increased the sensitivity to the alarmone to levels similar to those found for m*Inf*A (IC_50_ = 50 ± 5 μM) (Fig 6F). Pure in vitro translation of all m*Tuf*A variants using high ppGpp concentrations (2 mM) showed that SR III accounts for ppGpp tolerance (Fig 6G). Hence, SR III appears to be the minimal m*Tuf*A element promoting 30S IC formation and increasing GTP affinity for IF2, which in turn contributes to tolerate high concentrations of ppGpp and mRNA translation.

Altogether, our biochemical results indicate that SR III of m*Tuf*A accounts for increasing 30S IC formation, IF2 affinity gain for GTP, and promoting ppGpp tolerance. In addition, our bioinformatic analysis coincides with in vivo probing of mRNA structure, showing that SR III folds into consistent structures upstream of the TIR. Thus, SR III could be considered a structured enhancer of translation initiation (SETI).

### SETIs and the transition towards stringent response

The transition towards stringent response entails changes in the composition of all guanosine nucleotides. GTP and GDP concentrations decrease, while (p)ppGpp increase during stringent response. Our results demonstrate that the 30S IC responds to these varying stoichiometries of guanosine nucleotides in an mRNA-dependent manner. ppGpp and, to a lesser extent, GDP are able to negatively compete with GTP for IF2, while pppGpp can be used by the factor to promote translation initiation, albeit at an affinity and kinetic cost (Fig 2). To inquire how the 30S IC responds during the transition from non-stringent (NS) to stringent (S) guanosine nucleotide conditions in the cell, we measured translation initiation along 5 combinations of guanosine nucleotides by thermophoresis, from NS to S conditions (Fig 7A). NS conditions were set up to contain 1 mM GTP and 0.25 mM GDP, while S conditions contained 1 mM ppGpp, 0.25 mM pppGpp, 0.14 mM GTP, and 0.05 mM GDP. At NS conditions, 30S IC formation shows a trend similar to all of the above described results, with higher efficiencies for m*Tuf*A and followed by m*Inf*A and the model mMF1 (Fig 7A). All three mRNAs maintained their capacity to promote 30S IC formation during the first steps of the transition, indicating that the 30S IC can tolerate a rapid drop in GTP concentrations. Indeed, the *K*_*D*_ of GTP for the 30S-bound IF2 is lower than the nucleotide concentrations at NS or S conditions (Fig 3 and [31]). From the third step of the transition, concomitant to ppGpp building up, 30S IC formation decreased. However, the tolerance among mRNAs was maintained, with m*Tuf*A being the most tolerant to S guanosine nucleotide conditions (25% retained IC formation) (Fig 7A).

Our structural and biochemical analysis indicated that the m*Tuf*A SETI contributes to enhancing GTP affinity and ppGpp tolerance (Fig 6). m*Tuf*A lacking this structure showed low capacity of 30S IC formation at NS conditions and was more susceptible to ppGpp at S conditions (10% residual efficiency) (Fig 7B). On the other hand, m*Tuf*A constructs containing the SETI were able to promote efficient 30S ICs and were remarkably tolerant to (p)ppGpp accumulation at physiological stringent conditions of guanosine nucleotides (Fig 7B), allowing up to 50% initiation efficiency. To inquire whether the high initiation at S conditions was restricted to m*Tuf*A or could be exploited by other mRNAs, we modelled the UTR of RNA transcripts that were shown to be either regulated transcriptionally by ppGpp [39] or their corresponding protein abundancy increased in stationary as compared to exponential growth [33] (S2 File). From the 25 modeled UTRs, 12 show a similar secondary structure fold to that of the m*Tuf*A SETI, albeit their sequence differed greatly (S2 File). m*Rnr* was found among the up-regulated genes, contains a SETI, and codes for RNAse R. Two variants of the mRNA were produced, with and without the SETI element, and translation initiation was measured along the transition from NS to S conditions. m*Rnr*, containing the structured hairpins, promoted efficient 30S ICs and was remarkably tolerant to ppGpp accumulation at S conditions (Fig 7B), allowing up to 75% initiation efficiency, whereas m*Rnr* lacking the SETI element diminished its capability to promote 30S IC under both NS and S conditions (Fig 7B). The similar dependence of m*Tuf*A and m*Rnr* on the presence of the SETI to tolerate ppGpp indicates that this mechanism is not restricted to m*Tuf*A and can be exploited by other mRNAs.

Measurements of ppGpp tolerance for all mRNAs tested here indicate that in the absence of the SETI, the average tolerance was 7% ± 3%. ppGpp tolerance is defined here as the percentage of 30S ICs forming in the presence of a 4-fold excess of ppGpp over GTP as compared to the complexes formed in the absence of the alarmone (Fig 7C). Consistently, the presence of the SETI allowed a 2- to 7-fold increase in ppGpp tolerance, depending on the transcript, and reached up to 25% and 50% for m*Tuf*A and m*Rnr*, respectively (Fig 7C). In contrast, ppGpp tolerance of m*Tkt*B, up-regulated by ppGpp but lacking the SETI element, was low independently of the UTR length (Fig 7C). Remarkably, 3D modeling of the m*Rnr* SETI share a similar layout to that of m*Tuf*A (Fig 7D), suggesting that SETIs promote ppGpp tolerance by spatial or conformational constrains rather than through specific primary sequences.

## Discussion

**Fig 8:**
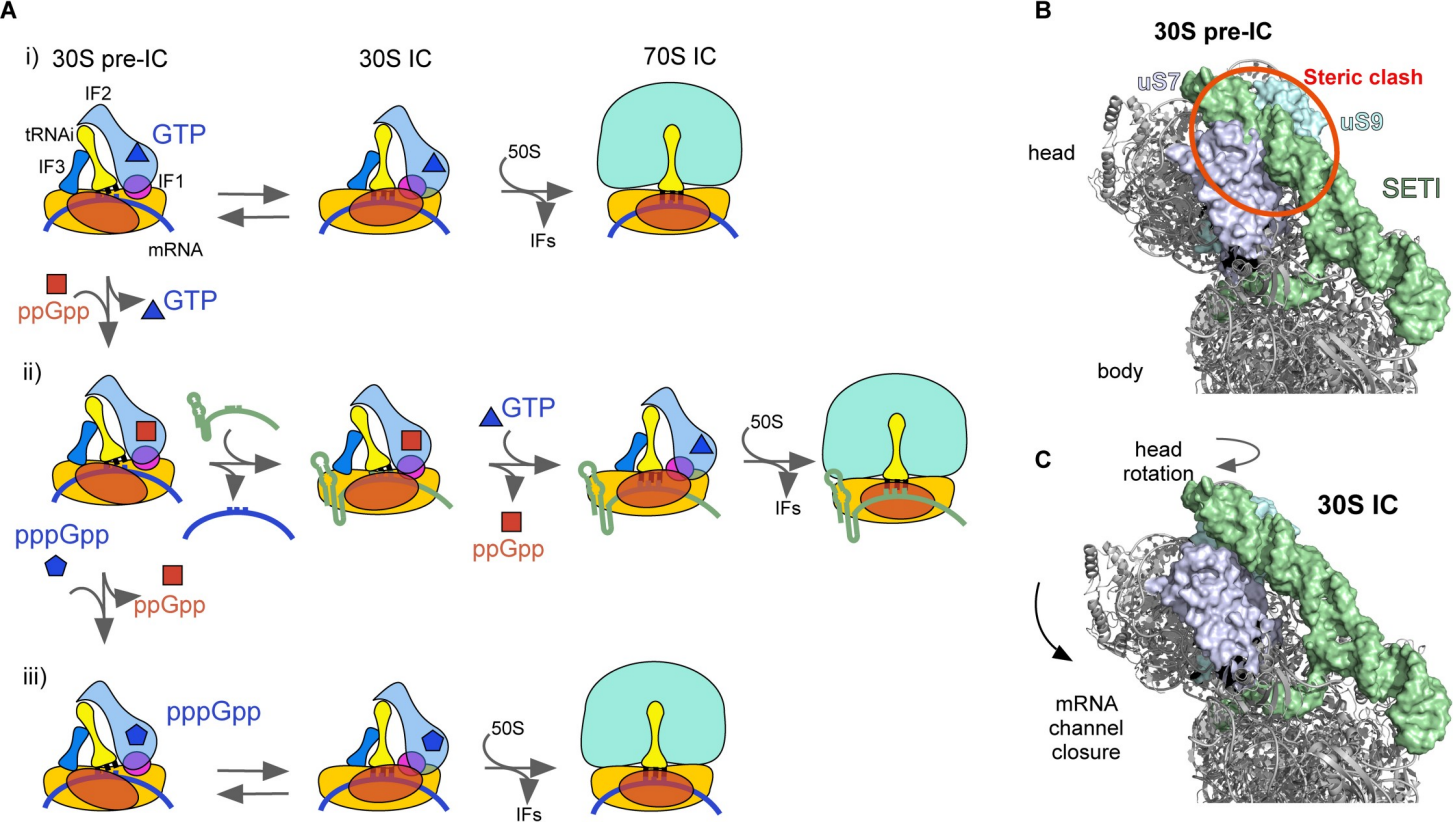
Model of translation initiation during stringent response. (A) Schematics representing translation initiation at NS conditions (i), at a high ppGpp/GTP ratio (ii), or by using pppGpp (iii) during stringent response. At high ppGpp/GTP ratio, ppGpp binds IF2, precluding start codon recognition and promoting the 30S pre-IC. In turn, the 30S pre-IC can exchange the bound mRNA for a more ppGpp-tolerable transcript, allowing GTP to replace the tetraphosphate and proceed to protein elongation. Alternatively, IF2 can bind pppGpp and proceed towards translation elongation. (B) Structural model of the 30S pre-IC programmed with m*Tuf*A SETI (green surface), structurally aligned on PDB 5LMP [42]. Ribosomal proteins uS7 (violet surface) and uS9 (cyan surface) are highlighted as potential sites for m*Tuf*A SETI interactions. The red circle indicates clashing of the m*Tuf*A SETI with the 30S pre-IC complex. (C) Structural model of the 30S IC (PDB 5LMV, [42]) and the m*Tuf*A SETI. Head movements related to 30S IC formation upon decoding the start codon and potentially enhanced by SETI elements are indicated by arrows [42,43]. GTP, guanosine triphosphate; IC, initiation complex; IF2, translation initiation factor IF2; m*Tuf*A, *Tuf*A mRNA; NS, non-stringent; PDB, Protein Data Bank; SETI, structured enhancer of translation initiation; tRNAi, initiator tRNA; uS7, universal ribosomal protein S7; uS9, universal ribosomal protein S9.

Our results provide unprecedented details on the dynamic regulation of the initiating ribosome and describe novel mechanisms that bacteria can use to cope with mRNA translation during stringent response. The canonical model suggests that upon (p)ppGpp accumulation, the translation machinery halts until more favorable growth conditions are available. A general slowdown of protein synthesis during stringent response was supported by several reports indicating ppGpp binds and inhibits translational GTPases [8,10]. However, the canonical model failed to explain how a subset of proteins are synthetized during overexpression of RelA, the primary (p)ppGpp synthetizing factor [40]. Additionally, (p)ppGpp activates the transcription of a number of genes. How these mRNAs are translated remained unexplained. More recent reports showed that (p)ppGpp are not restricted to stringent response, but their concentration fluctuates as a function of growth rate [19]. A tight RNA/protein and DNA/protein synthesis coordination in *E*. *coli* was shown to be regulated by (p)ppGpp [19]. On the other hand, the rate of protein elongation appears to be unaffected during stationary phase (starvation), characterized by high levels of the alarmone, indicating that elongating GTPases are not inhibited by (p)ppGpp [41]. Thus, (p)ppGpp-mediated inhibition of protein synthesis appears to be permissive rather than a strict on/off mechanism. Our results support a permissive mechanism, where IF2 translates the nutritional availability of the bacterial cell into protein synthesis efficiencies by sensing the ppGpp to GTP ratios and by using pppGpp (Fig 8A).

ppGpp accumulation results in IF2 modulating the 30S pre-IC to IC equilibrium, ultimately contributing to define the efficiency by which the mRNA enters the elongation phase of protein synthesis (Fig 8A). ppGpp-bound IF2 shifts the translation initiation equilibrium towards the liable 30S pre-IC in an mRNA-dependent manner. The potential of each mRNA to be translated arises from the intrinsic capability of mRNAs to program 30S ICs (Fig 3A), the GTP affinity for IF2-bound complexes (Fig 3C), and tolerance to ppGpp (Fig 3E). We observed that the m*Tuf*A mRNA is more tolerant to ppGpp than m*Inf*A despite that both mRNAs code for housekeeping and essential proteins (Fig 5). m*Tuf*A initiates translation 2- to 4-fold more efficiently than m*Inf*A in the absence of any competing mRNA. In a cellular context, the observed difference may be accentuated to the extent observed in vivo due to the availability of free 30S subunits, competing mRNAs, and transcriptional regulation [33,34]. On the other hand, the GTP affinity for 30S ICs programmed with m*Tuf*A is 7-fold higher if compared to m*Inf*A, both being in the low micromolar range. In contrast, previous studies reported two to three orders of magnitude lower affinities between free IF2 and GTP [8,28]. Thus, the formation of the 30S IC entails an affinity gain for GTP to the 30S-bound IF2 of up to two orders of magnitude, depending on the mRNA used (this study). Consequently, the affinity gain of the 30S-bound IF2 for GTP may allow ppGpp to compete differently for every given complex.

Our structural and biochemical analyses show that m*Tuf*A contains a SETI upstream of the SD-aSD duplex that largely accounts for the functional gains of the transcript. The m*Tuf*A SETI is composed by a dual hairpin element that is conserved among enterobacteria (S1 File), shows similar folding independently of the used algorithm, and is present in vivo (Fig 6A) [38]. Additionally, we show that the presence of the dual hairpin element is not only restricted to the m*Tuf*A UTR. Similar structures were found in other mRNAs, albeit showing low sequence identity (S2 File). Comparisons between SETIs of m*Tuf*A and m*Rnr* show that the removal of the structured hairpins leads to a 2- to 7-fold loss of ppGpp tolerance, reducing their capacity to assemble 30S ICs to similar levels seen in mRNAs that naturally lack the structured element (under 10%) (Fig 7C). Three-dimensional prediction and structural alignment using available 30S complexes locate the m*Tuf*A SETI on the head, between ribosomal proteins uS7 and uS9, and shoulder of the 30S subunit (Fig 8B). Comparison of the 30S pre-IC with the IC structures indicates that the SETI element could clash the head on the 30S pre-IC but not in the 30S IC. Thus, the SETI could promote a 30S conformation similar to that of the 30S IC (Fig 8C).

Cryo-electron microscopy reconstructions, single molecule, and biochemical assays suggest that the 30S subunit encounters conformational changes upon decoding the start codon on the mRNA (30S IC formation) [18,42–44]. Among these, the head rotates towards the A site and closes towards the body. These 30S movements are accompanied by the initiator tRNA, with its aminoacyl end moving towards the A site [42,43]. On the other hand, IF2 interacts with the tRNA through the C2 domain and contributes to the tRNA accommodation. Indeed, single molecule studies showed that initiator tRNA stabilizes the active conformation of IF2, which in turn may cooperatively enhance GTP binding ([18] and this study). Thus, molecular communications along IF2 gate the guanosine binding domain with the tRNA positioning, which in turn occurs concomitantly with movements of the 30S head [42]. Mutations in the C1 domain of IF2 disrupt the IF2 structural communication by promoting a GTP-like configuration in the presence of GDP and stabilizing fMet-tRNA^fMet^ on the 30S IC [18]. Conversely, a mutation on the G3 domain of the factor appeared to promote a GDP-like configuration, ultimately allowing IF2 dissociation in the absence of GTP hydrolysis [17]. Altogether, the SETI may contribute to a cascade of conformational events by promoting a 30S conformation that propagates through initiator tRNA and IF2 to the guanosine binding domain of the factor to signal the 30S IC to tolerate ppGpp.

During stringent response, cellular pppGpp increases together with the tetraphosphate, albeit reaching lower concentrations [31]. To our knowledge, this is the first study showing that IF2 can use the guanosine pentaphosphate, as an alternative mechanism by which the bacterial cell can initiate translation during stringent response. pppGpp can promote 30S IC, although at increased IF2 concentrations (Fig 2A). Formation of 70S pre-IC, as measured by 50S joining to 30S ICs, is unaffected (Fig 2C), whereas initiator tRNA accommodation is 2-fold slower if compared to the GTP-bound IF2 (Fig 2D). Both the higher IF2 concentration requirements during 30S IC formation and the slowed tRNA accommodation indicate that pppGpp induces yet a different conformation on IF2. Nevertheless, pppGpp can confer the initiating ribosome an alternative pathway to translate mRNAs (Fig 8A). The contribution of pppGpp-mediated translation initiation may be hindered due to high ppGpp concentrations under stringent response and/or by the coded mRNA. Indeed, our results indicate that the transition towards stringent response conditions entails a reduction of translation initiation efficiency even in the presence of the pentaphosphate (Fig 7). These variations are rather unaffected by GTP shortage due to its high affinity for IF2. However, 30S IC inhibition correlates with ppGpp accumulation in an mRNA-dependent manner (Fig 7A) by the presence of a SETI element at the 5′ UTR (Fig 7B). Thus, basal concentrations of GTP and accumulation of pppGpp can provide IF2 with the conformational cofactors to initiate protein synthesis.

Altogether, our study (i) confirms that IF2 plays a primary role for ppGpp-mediated inhibition of translation, (ii) shows that ppGpp-bound IF2 precludes the formation of the 30S IC rather than subsequent steps, (iii) indicates that the extent of ppGpp tolerance depends on the mRNA, (iv) shows that m*Tuf*A and m*Rnr* use a SETI to tolerate high ppGpp concentration, and (v) shows that IF2 can use pppGpp to initiate protein synthesis. These findings provide a novel rationale to explain how activated genes are ultimately translated into proteins during stringent response and broaden our knowledge of the regulatory mechanisms that bacteria could use to adapt to new environments.

## Material and methods

### Biological preparations

#### Ribosomal subunits

The 30S subunits were prepared from purified 70S ribosomes by sucrose gradient centrifugation in a zonal rotor (Ti-15, Beckman, CA) under dissociating conditions using buffer TAKM_3.5_ (50 mM Tris [pH 7.5], 70 mM NH_4_Cl, 30 mM KCl, and 3.5 mM MgCl_2_), essentially as described elsewhere [29]. Briefly, fractions containing 30S or 50S subunits were collected and pelleted in a Ti50.2 rotor at 50,000 rpm over 12 hours. The resulting 30S pellets were resuspended in buffer TAKM_7_ (as TAKM_3.5_ but containing 7 mM MgCl_2_). The concentration of 30S subunits was determined by measuring the absorbance at 260 nm using an extinction coefficient of 63 pmol/AU_260 nm_.

#### IFs

Cells harboring pET 21 (*Kan* resistant) expression plasmids with cloned either *infA*, *infB*, or *infC* (coding for IF1, 2, and 3, respectively) were grown in 6 L of LB medium supplemented with 30 μg/mL of kanamycin at 37 ˚C until they reached an optical density of 0.8 OD_600_. A final concentration of 1 mM of IPTG was added to induce protein expression, allowing expression for 3 hours. Cells were collected by centrifugation at 6,000 RCF and resuspended in buffer A (50 mM Tris-HCl [pH 7.1] and 5% v/v glycerol) with 200 mM KCl. 5 mM 2-mercaptoethanol, a protease tablet cocktail inhibitor (11836153001, Roche, Basel, Switzerland), 0.5 mM Pefabloc (11429868001, Roche, Basel, Switzerland), 1 mg/mL of lysozyme, and few crystals of DNAse (DN25, Sigma-Aldrich) were added to the ice-cold suspension of unfrozen cells. Lysates were obtained using a Misonix 3000 sonicator (EW-04711-81, Misonix, Newtown, Conneticut) for 5 minutes with 25% amplitude for 10 seconds sonication followed by 20 seconds of pausing to avoid overheating. The lysate was centrifuged in a JA30.5 rotor for 30 minutes at 25,000 rpm to remove cell debris. To dissociate IFs from the ribosomes, the concentration of KCl of the supernatant was increased to 0.7 M subsequently, and ribosomes were sedimented by centrifugation in a Ti50.2 rotor for 2 hours at 50,000 rpm, enriching the IFs in the supernatant.

Supernatants containing either IF1 or IF3 were diluted with buffer A to reach a final concentration of 0.1 M KCl. A HiTrap SP HP column (5 mL) (17-1151-01, GE Healthcare, Chicago, Illinois) was equilibrated with buffer A containing 0.1 M KCl prior to loading the clarified lysates. IF1 was eluted with a linear gradient of 0.05 to 1 M KCl in buffer A. Fractions containing IF1 were identified using 18% acrylamide SDS-PAGE, pooled, and concentrated using an Amicon centrifugation membrane with a 3-kDa cutoff (UFC900308, Merck, Darmstadt, Germany). Size exclusion using a HiLoad 26/60 Superdex 75 prep grade column (17-1070-01, GE Healthcare, Darmstadt, Germany) was necessary to further purify IF1 from contaminants of higher molecular weight. Typically, 0.5 mL of IF1 was loaded, separated with a flow rate of 1 mL/minute, and elution was monitored by absorbance at 290 nm. IF3 was purified similarly to IF1 using a cation exchange chromatography on a HiPrep CM FF 16/10 column (28-9365-42, GE Healthcare, Chicago, Illinois), however, using a stronger gradient in Buffer A (0.1 to 1 M NH_4_Cl). IF3 elutes with very high purity, as observed using 15% SDS-PAGE.

Cell lysates containing IF2 were processed essentially as described for IF1 with the following modifications. Affinity chromatography on Hi-Trap His Column (54835, GE Healthcare, Chicago, Illinois) was used as a first step as a 6×His-tag was added at the amino terminal domain of IF2. A 50 to 300 mM imidazole gradient in Buffer A containing 300 mM KCl was used to elute IF2. Fractions containing the factor were pooled together and dialyzed overnight to buffer A containing 50 mM KCl prior to cation exchange chromatography. A 50–500 mM KCl gradient was used to elute IF2 from a 5-mL Hi-Trap SP HP column (17115201, GE Healthcare, Chicago, Illinois). Fractions were analyzed by 8% acrylamide SDS-PAGE and those containing IF2 were pooled together. All three IFs were finally dialyzed in Storage Buffer (50 mM Tris [pH 7.1], 200 mM NH_4_Cl, 5 mM 2-mercaptoethanol, and 10% v/v glycerol); small aliquots were flash frozen in liquid nitrogen and stored at −80 ˚C.

#### Bpy-Met-tRNA^fMet^ (Bpy-tRNAi)

Met-tRNA^fMet^ was prepared essentially as described elsewhere [45]. NHS ester BODIPY FL SSE dye (D6140, Invitrogen, Carlsbad, California, or analogous) was used to label Met-tRNA^fMet^ at the amino group of the amino acid as follows. Met-tRNA^fMet^ was incubated in 50 mM HEPES-KOH (pH 8.5) with 3 mM of the dye in the dark. The reaction was stopped by adding 0.3 M potassium acetate (pH 5.0). Bpy-Met-tRNA^fMet^ was purified by three sequential ethanol precipitations and HPLC chromatography on a reverse phase C18 column with a 5%–40% ethanol gradient. The efficiency of labelling was determined by the molar stoichiometry of the compound measuring both tRNA and dye absorbance.

#### mRNAs

DNA templates for mRNAs were amplified by PCR using the Maxima Hot Start Green PCR Master Mix (K1062, ThermoScientific, Waltham, Massachusetts) and corresponding primers (S1 Table). Essentially, the reaction contained 20–200 ng of DNA template, 1 μM of each primer, Master Mix (containing Buffer, NTPs, and polymerase), and deionized water. Primers were synthetized by Macrogen (South Korea). PCR products were purified using the GeneJET PCR Purification Kit (K0702, ThermoScientific, Waltham, Massachusetts) (S2 Table). mRNAs were produced by in vitro transcription for 3 hours at 37°C. The reaction contained Transcription Buffer (40 mM Tris-HCl [pH 7.5], 15 mM MgCl_2_, 2 mM spermidine, and 10 mM NaCl), 10 mM DL-Dithiothreitol (DTT), 2.5 mM NTPs, 5 mM guanosine monophosphate, 0.01 u/μL inorganic pyrophosphatase, 2 U/μL T7 polymerase, 5 ng/μL DNA template, and deionized water. Transcripts were then purified using Direct-Zol RNA MiniPrep (R2052, Zymo Research, Irvine, California) and visualized by 8 M urea PAGE followed by staining in Methylene blue (S3 Table).

#### Rel_Seq_

For preparation of ppGpp, we first purified the N-terminal fragment containing the 385 first amino acids of native Rel_Seq_ protein. The enzyme was purified from BL21 (DE3) cells transformed with pET21 plasmid encoding C-terminal 6×His-tagged fragment. Cells were grown in LB medium with 100 μg/mL ampicillin at 37°C to an 0.6 OD_600_, and protein expression was induced with 1 mM IPTG with additional incubation for 3 hours. Cells were collected by low-speed centrifugation (6,000 rpm × 20 minutes, JLA8.1 rotor). Cell pellets were resuspended in lysis buffer (20 mM Tris-HCl [pH 7.9], 300 mM KCl, 5 mM MgCl_2_, 20% glycerol, 10 mM imidazole, 1 mM DTT, 280 μg/mL lysozyme, 0.1 mg/mL DNase I [DN25, Sigma-Aldrich, St. Louis, Missouri], and 1 tablet of protease inhibitor cocktail [11836153001, Roche]). Cells were opened using the EmulsiFlex-C3 (Avestin) and the cell debris was removed by centrifugation (45,000 rpm × 30 minutes, rotor Ti50.2). The supernatant was applied to a HisTrap FF (5 mL) column (17531901, GE Healthcare, Chicago, Illinois) for affinity chromatography. The column was washed with buffer HT (20 mM Tris-HCl [pH 7.9], 5 mM MgCl_2_, 20% glycerol, and 1 mM DTT) with 300 mM KCl and 10 mM imidazole, and the protein was eluted by a linear gradient from 10 mM to 200 mM imidazole in the same buffer. The eluted product was dialyzed twice against the buffer HT and 450 mM KCl. The protein was concentrated by Amicon Ultra-15 Centrifugal Filters (10 kDa) (UFC901008, Merck) and diluted in buffer HT with 50 mM KCl to decrease KCl concentration to 90 mM. An anion-exchange chromatography step was used to further purify Rel_Seq_. HiTrapQ (5 mL) column (17115301, GE Healthcare, Chicago, Illinois) was equilibrated with the buffer (20 mM Tris-HCl [pH 9.5], 50 mM KCl, 5 mM MgCl_2_, 20% glycerol, 1 mM DTT), and the protein was eluted by a linear gradient from 50 mM to 750 mM KCl. Fractions containing Rel_Seq_ were analyzed by 12% SDS-PAGE gel-electrophoresis, pooled, aliquoted, and frozen in liquid nitrogen. The enzyme activity of Rel_Seq_ was above 80%, as measured by ppGpp production over the sum of ppGpp plus GDP.

#### (p)ppGpp

Preparative synthesis of the tetraphosphate and pentaphosphate were performed in buffer B (30 mM Tris-HCl [pH 8.0], 100 mM NaCl, 10 mM MgCl_2_) using 10 mM ATP, 4 mM GDP or GTP, and 50 μM Rel_Seq_. The reaction was incubated for 40 minutes at 37°C and stopped by phenol extraction. The water phase was loaded to a MonoQ 5/50 GL (1 mL) column (GE17-5166-01, GE Healthcare, Chicago, Illinois). (p)ppGpp eluted with a linear gradient from 0.5 mM to 600 mM LiCl in buffer C (25 mM Tris-HCl [pH 8.3], 0.5 mM EDTA). Fractions containing (p)ppGpp were pooled and precipitated with 1.5 M LiCl and 2 volumes of absolute ethanol. The precipitate was harvested by centrifugation (16,100 RFC × 20 minutes) and dissolved in water. (p)ppGpp concentration was measured by UV absorbance at 253 nm using an extinction coefficient of 13,700. Samples were aliquoted and stored at −20°C.

### Experimental conditions and analysis

The 30S complexes were reconstituted using pure subunits, IFs, tRNAi, mRNA, and nucleotides. The 30S subunits were reactivated by incubation in TAKM_20_ buffer (as TAKM_3.5_ but with 20 mM MgCl_2_) for 1 hour at 37°C prior to use. Generally, 30S ICs were formed by incubation at 37°C for 30 minutes in TAKM_7_ using 1 μM 30S subunits, 4 μM mRNA, 2 μM IF1, 1 μM IF2, 1.5 μM IF3, 0.5 μM Bpy-Met-tRNA^fMet^, and 0.2 mM guanosine nucleotides, unless otherwise stated in the Results or figure captions. Ligand titrations were prepared using serial dilutions using 1:1 mixing of complexes containing the highest concentration of the titrant with complexes lacking only the ligand under investigation. The number of reactions were set up to cover a wide range of concentrations.

MST was measured on 10-μL reactions using standard capillaries (MO-K022, NanoTemper Technologies, Munich, Germany) on a Monolith NT.115 (NanoTemper Technologies, Munich, Germany) at monochromatic LED with power input of 30% and IR laser power of 40%. The local temperature perturbation was expected to be less than 3°C. All measurements were performed at room temperature (22 ± 2°C). Three to four replicates were measured for each investigated reaction to calculate mean and standard deviation. Day to day reproducibility was very high, which allowed to report absolute thermophoresis values rather than to use normalized MST traces. *K*_*D*_ were calculated using a hyperbolic or quadratic function using Graphpad Prism 6.0 (GraphPad, San Diego, California) or software provided by NanoTemper. Same-site inhibitory constants were calculated using the *K*_*D*_ values obtained for GTP and concentrations used by nonlinear fitting using a same-site competition function using Graphpad Prism 6.0 (GraphPad, San Diego, California).

To measure 70S pre-IC formation, a SX-20 stopped-flow instrument (Applied Photophysics, Leatherhead, UK) was set up for LS recording with an excitation wavelength of 430 nm. Scattered light was measured at an angle of 90° without a filter. Pseudo first-order conditions were approximated by using ≥3-fold excess of 50S subunits over 30S ICs; 0.2 μM 30S ICs were rapidly mixed in the instrument with 0.6 μM 50S at 25 ˚C. Bpy-tRNAi accommodation was measured essentially as described elsewhere [14]. Briefly, 0.2 μM 30S ICs were formed using Bpy-tRNAi and rapidly mixed with 0.6 μM 50S at 25 ˚C. Fluorescence was excited at 470 nm and measured after a 495-nm longpass filter. Individual traces (6–11 traces) were averaged, and the resulting kinetic curve was approximated by single (F = A_0_ + A_1_*exp (-*k*_app1_*t)) or double (F = A_0_ + A_1_*exp (-*k*_app1_*t) + A_2_*exp (-*k*_app2_*t)) exponential fit using Graphpad Prism 6.0 software (GraphPad, San Diego, California).

In order to visualize overall protein synthesis, we used a coupled transcription-translation cell-free system. Reactions were carried out in 10 μL of the purified components from the PURExpress *In Vitro* Protein Synthesis Kit (E6800S/L, NEB, Ipswich, Massachusetts) with the corresponding templates. These reactions were incubated at 37°C for 2 hours. To assess ppGpp inhibitory capacity in translation, 0.15 μM DNA or 1 μM mRNA, coding for m*Tuf*A or m*Inf*A, was introduced in the described system in the absence or presence of 2 mM ppGpp or at varying alarmone concentration. After incubation, sample processing and fluorescent labelling were carried out as described by the Lumio Green Detection Kit (LC6090, ThermoScientific, Waltham, Massachusetts) for in vitro reactions. Finally, 20 μL of labelled samples were loaded into 20% SDS-PAGE gels and visualized under a Blue-light LED transilluminator with orange filter (Cleaver Scientific LTD, Warwickshire, UK). Gel quantification was performed by pixel densitometry analysis using ImageJ software [37].

### Structural modelling of mRNAs

The secondary structure of the complete mRNAs and its variants were predicted using the RNAfold [46] and mFOLD servers [47]. We used the default server conditions and simulated the 30S-bound structural layout by incorporating unpaired constrains from the SD to the start of the coding sequence (S1 File). To model the in vivo secondary structure, unpaired nucleotides resulting from chemical probing were set as unbound constrains [38] (S1 File).

Three-dimensional structures of the mRNAs were built using the RNAcomposer server, and the resulting pdb file was visualized in Pymol and MOE software [48] (Chemical Computing Group, Montreal, Canada). Structural alignments and RMSD between two structures were calculated using the MOE software (Chemical Computing Group, Montreal, Canada). For the structural modelling of the 30S IC and pre-IC, the predicted SR IIIb and TIR until +20 from the GUG were aligned with the bound mRNA of PDBs 5lmp and 5lmv, respectively [42]. mRNA structural alignments and visualization of the resulting models were performed with Pymol software (Schrödinger, New York, New York). Alignment and secondary structure prediction of the m*Tuf*A SETI of *E*. *coli*, *Shigella dysenteriae*, and *Salmonella enterica* were performed using the PETfold web server (S1 File) [49].

## Supporting information

S1 Fig Step assignment for 30S IC formation by MST measurements (related to Fig 1C). Time dependencies of fluorescence measurements for Bpy-tRNAi interacting with 30S ribosomal complexes programmed with mMF1 containing AUG **(A)** or UUC **(B)** initiation codon. Time traces of Bpy-Phe-tRNA^Phe^ binding to 30S complexes using mMF1 containing AUG **(C)** or UUC **(D)** as initiation codons. Colors represent increasing concentrations of mRNA (from 2 nM to 4 μM). Bpy-Phe-tRNA^Phe^, Bodipy labelled Phe-tRNA^Phe^; Bpy-tRNAi, Bodipy labelled initiator tRNA; IC, initiation complex; MST, Microscale Thermophoresis.
(PDF)

S2 Fig IF2-Bpy-tRNAi complex formation as a function of guanosine nucleotides. **(A)** IF2 concentration dependencies for Bpy-tRNAi binding in the presence of 0.5 mM of the indicated guanosine nucleotides to build IF2-tRNA-GNP ternary complexes (TCs). **(B)** Comparison of the calculated *K*_*D*_ from interactions measured in (A) with a similar IF2 titration performed in the complete 30S IC complex with GDP or ppGpp and in the absence of any nucleotide (Apo) (Fig 2A). Three to five measurements were performed; mean and standard deviation are plotted (S1 Data). Bpy-tRNAi, Bodipy labelled initiator tRNA; GDP, guanosine diphosphate; GNP, guanosine nucleotide; IC, initiation complex; IF2, translation initiation factor IF2.
(PDF)

S3 Fig Formation of 30S IC as a function of the mRNA, model or natural. **(A)** Time courses of 30S IC formation for increasing concentrations of the model mMF1. **(B)** Time courses of m*Inf*A- or **(C)** m*Tuf*A-dependent 30S IC formation. Colors represent increasing concentrations of mRNA (from 2 nM to 4 μM). IC, initiation complex; m*Inf*A, *Inf*A mRNA; m*Tuf*A, *Tuf*A mRNA.
(PDF)

S4 Fig Inhibition of 30S IC formation by GDP or ppGpp competition with GTP (related to Fig 3). **(A)**, **(B)**, and **(C)** show time courses of thermophoresis for mMF1, m*Inf*A, and m*Tuf*A, respectively, at increasing concentrations of GDP competing with 50 μM GTP used for 30S IC formation. **(D)**, **(E)** and **(F)** show time courses of thermophoresis for mMF1, m*Inf*A, and m*Tuf*A, respectively, at increasing concentrations of ppGpp competing with 50 μM GTP used for 30S IC formation. **(G)** MST dependency as a function of the logarithm of GDP concentration. Symbols are as indicated, 3 measurements were performed, and mean and standard deviation are plotted (S1 Data). Continuous lines show nonlinear regression fittings with a same-site inhibition model. GDP, guanosine diphosphate; GTP, guanosine triphosphate; IC, initiation complex; m*Inf*A, *Inf*A mRNA; MST, Microscale Thermophoresis; m*Tuf*A, *Tuf*A mRNA.
(PDF)

S5 Fig Inhibitory effect of 70S pre-IC and 70S IC formation by GDP competing with GTP for IF2 (related to Fig 4). ICs (30S) containing 20 μM GTP and 200 μM GDP, programmed with mMF1 (blue), m*Inf*A (purple), or m*Tuf*A (red), were rapidly mixed with 50S subunits in a stopped-flow apparatus and scattered light **(A)** or Bpy-tRNAi fluorescence **(B)** were monitored over time as described in Fig 4. Seven to ten individual replicates were recorded and averaged. Nonlinear regression fitting with exponential functions is shown as continuous black lines. Bpy-tRNAi, Bodipy labelled initiator tRNA; GDP, guanosine diphoshate; GTP, guanosine triphosphate; IC, initiation complex; IF2, translation initiation factor IF2; m*Inf*A, *Inf*A mRNA; m*Tuf*A, *Tuf*A mRNA.
(PDF)

S6 Fig Guanosine nucleotide dependencies for 70S pre-IC formation under noncompetitive conditions (related to Fig 4). Time courses of 70S pre-ICs formation in the absence of any nucleotide (purple) or in the presence of 0.2 mM of either GDP (green) or ppGpp (orange). ICs (30S) were programmed with either mMF1 **(A)**, m*Inf*A **(B)**, or m*Tuf*A **(C)**. The 70S pre-IC was measured by scattered light with a stopped-flow apparatus. Seven to ten individual replicates were recorded and averaged. Nonlinear regression fitting is shown as continuous black lines. GDP, guanosine diphosphate; IC, initiation complex; m*Inf*A, *Inf*A mRNA; m*Tuf*A, *Tuf*A mRNA.
(PDF)

S7 Fig Guanosine nucleotide dependencies for 70S IC formation as monitored by Bpy-tRNAi accommodation under noncompetitive conditions (related to Fig 4). Time courses of 70S ICs formation in the absence of any nucleotide (purple) or in the presence of 0.2 mM of either GDP (green) or ppGpp (orange). ICs (30S) were programmed with either mMF1 **(A)**, m*Inf*A **(B)**, or m*Tuf*A **(C)**. IC (70S) was measured by fluorescence change of Bpy-tRNAi with a stopped-flow apparatus. Seven to ten individual replicates were recorded and averaged. Nonlinear regression fitting is shown as continuous black lines. Bpy-tRNAi, Bodipy labelled initiator tRNA; GDP, guanosine diphosphate; IC, initiation complex; m*Inf*A, *Inf*A mRNA; m*Tuf*A, *Tuf*A mRNA.
(PDF)

S8 Fig In vitro translation of mTufA truncations (related to Fig 6) in the presence or absence of 2 mM ppGpp. A 20% SDS-PAGE was used to resolve the Lumio-tagged EF-Tu peptides (first 33 amino acids) and further visualized under a Blue-light transilluminator with a 530-nm filter. ImageJ software was used to determine pixel densities for each band over 3 different pictures. EF-Tu, translation elongation factor thermounstable; m*Tuf*A, *Tuf*A mRNA.
(PDF)

S1 Table Primer sequences for DNA template amplification. Primer sequences for DNA template amplification. *Tuf*A and *Inf*A are naturally encoded in the *E*. *coli* genome, while the MF1 coding sequence was cloned in a pTZ18R plasmid. Primers for MF1 correspond to the T7 promoter and terminator sequences. The sequence coding for a tetracysteine motif, added as an overhang sequence to the reverse primer, is indicated with the L suffix. A T7 promoter overhang was added to the forward primer to allow in vitro transcription. *Inf*A, *Inf*A gene; MF1, model gene derived from the 022 construct; *Tuf*A, *Tuf*A gene; T7, bacteriophage T7.
(DOCX)

S2 Table Gene sequences used for mRNA synthesis. Gene sequences used for mRNA synthesis. A T7 promoter was added by PCR for all natural mRNAs, while mMF1 encoded the promoter in the plasmid. Blue sequences indicate 5′ UTR. Yellow sequences indicate the coding region. Lowercase indicates the start codon. Underlined sequences indicate priming regions for PCR amplifications (S1 Table). A PCR amplification of the template p022 generates a short coding region for in vitro transcription, here called mMF1. T7, bacteriophage T7.
(DOCX)

S3 Table mRNA sequences for MST and in vitro translation analysis. mRNA sequences for MST and in vitro translation (L) analysis. Lowercase indicates the start codon. For mMF1, parenthesis indicate alternative start codons. MST, Microscale Thermophoresis.
(DOCX)

S1 File Excel spreadsheet containing secondary structure comparisons for *Tuf*A UTRs. Sheet 1 (m*Tuf*A_SR): Secondary structure predictions for each structured region within the *Tuf*A UTR. Sheet 2 (m*Tuf*A): Secondary structure prediction for the m*Tuf*A complete sequence. Sheet 3 (Enterobacteria): Alignment and secondary structure prediction of the -100 UTR of *Tuf*A from enterobacteria performed with the PETfold web server. m*Tuf*A, *Tuf*A mRNA; SR, structured region.
(XLSX)

S2 File Excel spreadsheet of mRNAs with and without SETI elements. **Sequence alignments and secondary structure comparisons of the m*Tuf*A with the m*Rnr* SETIs.** Sheet 1 (SETIs screening): Summary table for a number of *E*. *coli* genes with and without SETIs. Sheet 2 (m*Tuf*A and m*Rnr* alignment): Secondary structure and sequence comparison between the SR IIIb regions from m*Tuf*A and m*Rnr*. m*Rnr*, *Rnr* mRNA; m*Tuf*A, *Tuf*A mRNA; SR, structured region; SETI, structured enhancer of translation initiation.
(XLSX)

S1 Data Excel spreadsheet containing the individual quantitative observations that underlie the data summarized in the figures and results. Excel sheets are named accordingly to figures, indicating the respective panel.
(XLSX)

## Abbreviations

Bpy-tRNAi: Bodipy labelled tRNAi
DMS: dimethyl sulfide
FL: full-length
GDP: guanosine diphosphate
GTP: guanosine triphosphate
IC: initiation complex
IF: initiation factor
LS: light scattering
m*Inf*A: *Inf*A mRNA
MST: Microscale Thermophoresis
m*Tuf*A: *Tuf*A mRNA
m*Rnr*: *Rnr* mRNA
NS: non-stringent
RMSD: root mean square deviation
S: stringent
SD: Shine-Dalgarno
SETI: structured enhancer of translation initiation
SR: structured region
TE: translation efficiency
TIR: translation initiation region
tRNAi: initiator transfer RNA (fMet-tRNA^fMet^ in bacteria)
