# Supplementary figures and images for: How the initiating ribosome copes with ppGpp to translate mRNAs
Source: PLoS Biol. 2020 Jan 29;18(1):e3000593. doi: 10.1371/journal.pbio.3000593 (PMC7010297; doi:10.1371/journal.pbio.3000593)

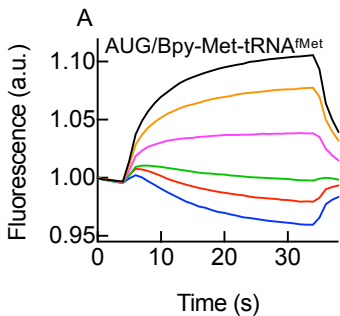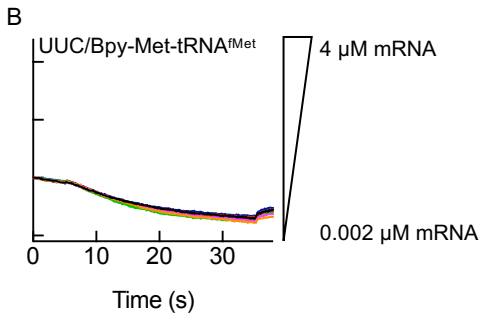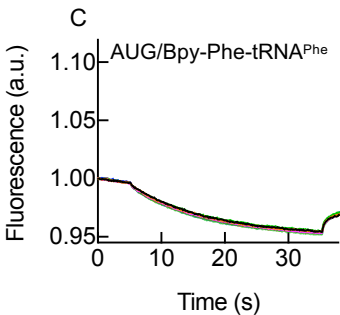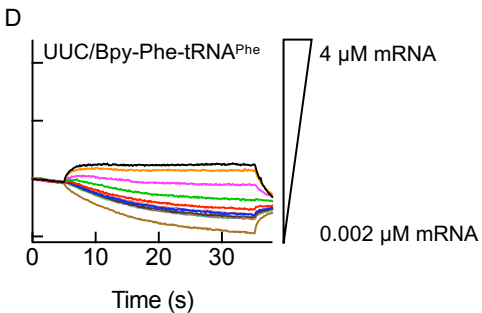

Supplement: S1 Fig — Time dependencies of fluorescence measurements for Bpy-tRNAi interacting with 30S ribosomal complexes programmed with mMF1 containing AUG (A) or UUC (B) initiation codon. Time traces of Bpy-Phe-tRNAPhe binding to 30S complexes using mMF1 containing AUG (C) or UUC (D) as initiation codons. Colors represent increasing concentrations of mRNA (from 2 nM to 4 μM). Bpy-Phe-tRNAPhe, Bodipy labelled Phe-tRNAPhe; Bpy-tRNAi, Bodipy labelled initiator tRNA; IC, initiation complex; MST, Microscale Thermophoresis. (PDF) [file pbio.3000593.s001.pdf]

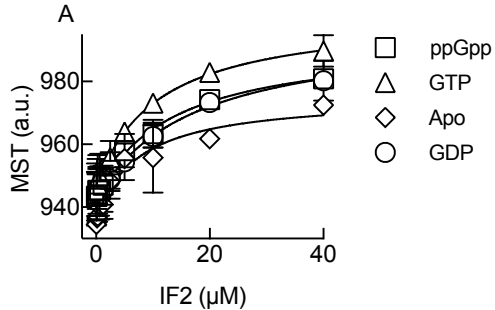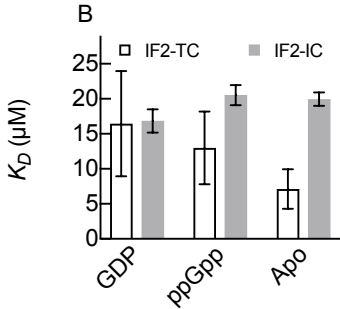

Supplement: S2 Fig — (A) IF2 concentration dependencies for Bpy-tRNAi binding in the presence of 0.5 mM of the indicated guanosine nucleotides to build IF2-tRNA-GNP ternary complexes (TCs). (B) Comparison of the calculated KD from interactions measured in (A) with a similar IF2 titration performed in the complete 30S IC complex with GDP or ppGpp and in the absence of any nucleotide (Apo) (Fig 2A). Three to five measurements were performed; mean and standard deviation are plotted (S1 Data). Bpy-tRNAi, Bodipy labelled initiator tRNA; GDP, guanosine diphosphate; GNP, guanosine nucleotide; IC, initiation complex; IF2, translation initiation factor IF2. (PDF) [file pbio.3000593.s002.pdf]

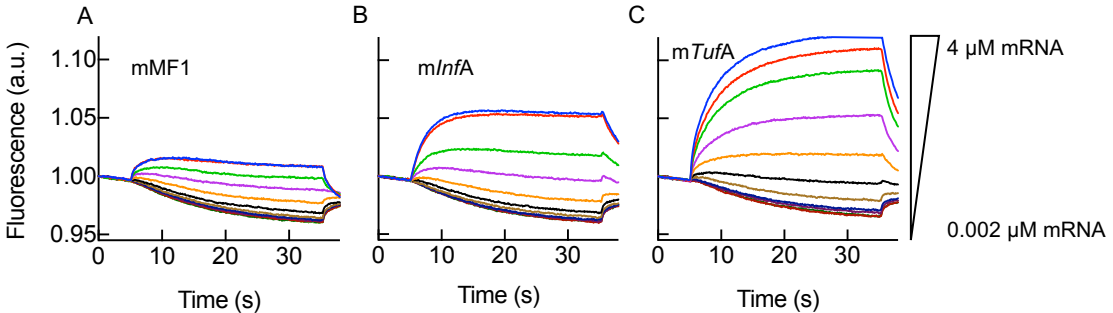

Supplement: S3 Fig — (A) Time courses of 30S IC formation for increasing concentrations of the model mMF1. (B) Time courses of mInfA- or (C) mTufA-dependent 30S IC formation. Colors represent increasing concentrations of mRNA (from 2 nM to 4 μM). IC, initiation complex; mInfA, InfA mRNA; mTufA, TufA mRNA. (PDF) [file pbio.3000593.s003.pdf]

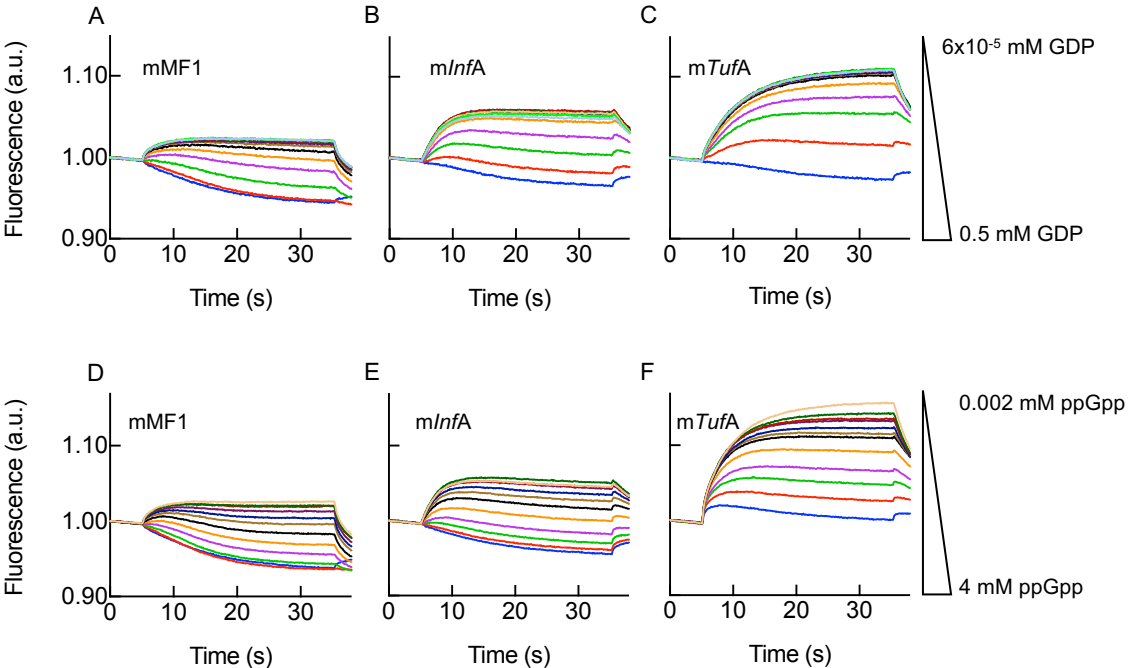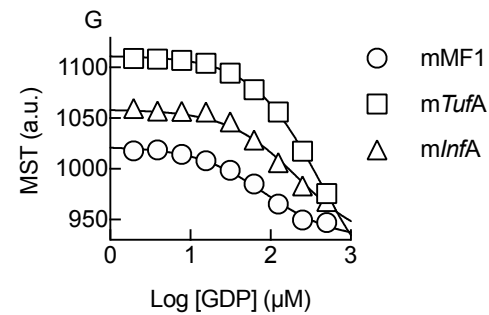

Supplement: S4 Fig — (A), (B), and (C) show time courses of thermophoresis for mMF1, mInfA, and mTufA, respectively, at increasing concentrations of GDP competing with 50 μM GTP used for 30S IC formation. (D), (E) and (F) show time courses of thermophoresis for mMF1, mInfA, and mTufA, respectively, at increasing concentrations of ppGpp competing with 50 μM GTP used for 30S IC formation. (G) MST dependency as a function of the logarithm of GDP concentration. Symbols are as indicated, 3 measurements were performed, and mean and standard deviation are plotted (S1 Data). Continuous lines show nonlinear regression fittings with a same-site inhibition model. GDP, guanosine diphosphate; GTP, guanosine triphosphate; IC, initiation complex; mInfA, InfA mRNA; MST, Microscale Thermophoresis; mTufA, TufA mRNA. (PDF) [file pbio.3000593.s004.pdf]

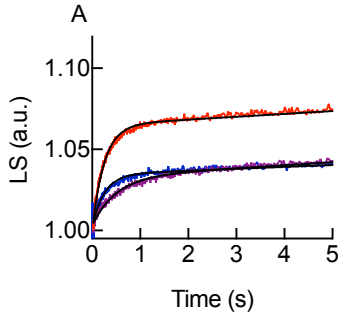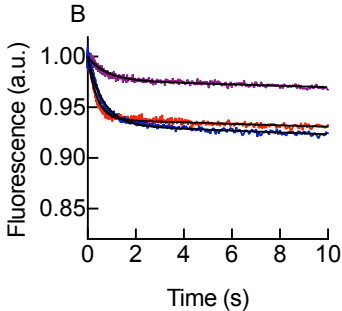

Supplement: S5 Fig — ICs (30S) containing 20 μM GTP and 200 μM GDP, programmed with mMF1 (blue), mInfA (purple), or mTufA (red), were rapidly mixed with 50S subunits in a stopped-flow apparatus and scattered light (A) or Bpy-tRNAi fluorescence (B) were monitored over time as described in Fig 4. Seven to ten individual replicates were recorded and averaged. Nonlinear regression fitting with exponential functions is shown as continuous black lines. Bpy-tRNAi, Bodipy labelled initiator tRNA; GDP, guanosine diphoshate; GTP, guanosine triphosphate; IC, initiation complex; IF2, translation initiation factor IF2; mInfA, InfA mRNA; mTufA, TufA mRNA. (PDF) [file pbio.3000593.s005.pdf]

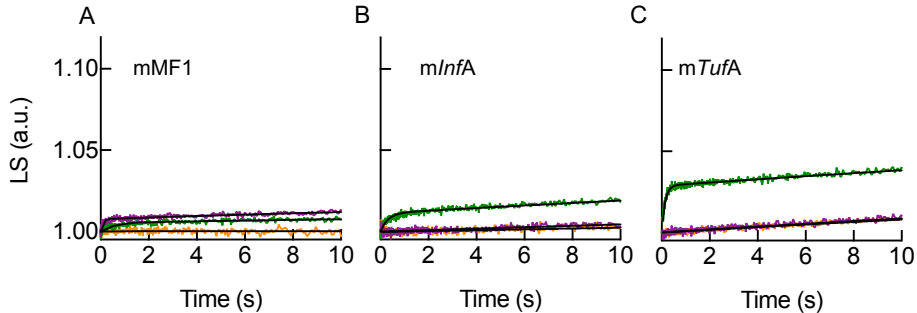

Supplement: S6 Fig — Time courses of 70S pre-ICs formation in the absence of any nucleotide (purple) or in the presence of 0.2 mM of either GDP (green) or ppGpp (orange). ICs (30S) were programmed with either mMF1 (A), mInfA (B), or mTufA (C). The 70S pre-IC was measured by scattered light with a stopped-flow apparatus. Seven to ten individual replicates were recorded and averaged. Nonlinear regression fitting is shown as continuous black lines. GDP, guanosine diphosphate; IC, initiation complex; mInfA, InfA mRNA; mTufA, TufA mRNA. (PDF) [file pbio.3000593.s006.pdf]

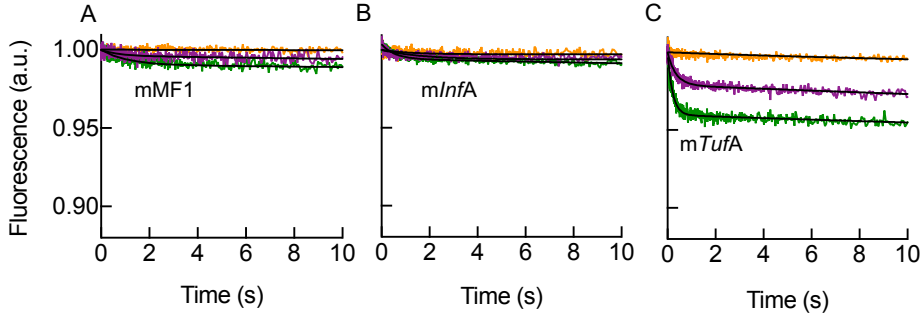

Supplement: S7 Fig — Time courses of 70S ICs formation in the absence of any nucleotide (purple) or in the presence of 0.2 mM of either GDP (green) or ppGpp (orange). ICs (30S) were programmed with either mMF1 (A), mInfA (B), or mTufA (C). IC (70S) was measured by fluorescence change of Bpy-tRNAi with a stopped-flow apparatus. Seven to ten individual replicates were recorded and averaged. Nonlinear regression fitting is shown as continuous black lines. Bpy-tRNAi, Bodipy labelled initiator tRNA; GDP, guanosine diphosphate; IC, initiation complex; mInfA, InfA mRNA; mTufA, TufA mRNA. (PDF) [file pbio.3000593.s007.pdf]

2 mM  
ppGpp

*wt mTufA*

+100

+200

+371

-

+

-

+

-

+

-

+

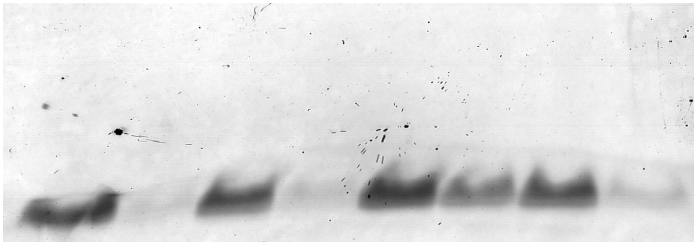

Supplement: S8 Fig — A 20% SDS-PAGE was used to resolve the Lumio-tagged EF-Tu peptides (first 33 amino acids) and further visualized under a Blue-light transilluminator with a 530-nm filter. ImageJ software was used to determine pixel densities for each band over 3 different pictures. EF-Tu, translation elongation factor thermounstable; mTufA, TufA mRNA. (PDF) [file pbio.3000593.s008.pdf]
