# Supplementary material for: How the initiating ribosome copes with ppGpp to translate mRNAs
Source: PLoS Biol. 2020 Jan 29;18(1):e3000593. doi: 10.1371/journal.pbio.3000593 (PMC7010297; doi:10.1371/journal.pbio.3000593)
Supplement: S1 Table — Primer sequences for DNA template amplification. TufA and InfA are naturally encoded in the E. coli genome, while the MF1 coding sequence was cloned in a pTZ18R plasmid. Primers for MF1 correspond to the T7 promoter and terminator sequences. The sequence coding for a tetracysteine motif, added as an overhang sequence to the reverse primer, is indicated with the L suffix. A T7 promoter overhang was added to the forward primer to allow in vitro transcription. InfA, InfA gene; MF1, model gene derived from the 022 construct; TufA, TufA gene; T7, bacteriophage T7. (DOCX) [file pbio.3000593.s009.docx]

**S1 Table**

Primer sequences for DNA template amplification. *Tuf*A and *Inf*A are naturally encoded in the *E. coli* genome while MF1 coding sequence was cloned in a pTZ18R plasmid. Primers for MF1 correspond to the T7 promoter and terminator sequences. The sequence coding for a tetra cysteine motif, added as an overhang sequence to the reverse primer, is indicated with the L suffix. A T7 promoter overhang was added to the forward primer to allow *in vitro* transcription.

| Name | Forward primer (5’🡪3’) | Reverse primer (5’🡪3’) | DNA (bp) | mRNA (nt) |
| --- | --- | --- | --- | --- |
| TufA | TAATACGACTCACTATAGGTTCTATCGCCTTTAAAGAAGGC | GCATGTTAGGTGATTGCAGCGGTCAGAG | 514 | 497 |
| TufA_L | TAATACGACTCACTATAGGTTCTATCGCCTTTAAAGAAGGC | GCATGTTAGCAGCAGCCCGGGCAGCAGGTGATTGCAGCGGTCAGAG | 540 | 523 |
| TufA_100_L | TAATACGACTCACTATAGGGACGTTATCGGTGACTTGAGCCGTCG | GCATGTTAGCAGCAGCCCGGGCAGCAGGTGATTGCAGCGGTCAGAG | 440 | 423 |
| TufA_200_L | TAATACGACTCACTATAGGTGTTCGGATACGCAACTCAGCTGCG | GCATGTTAGCAGCAGCCCGGGCAGCAGGTGATTGCAGCGGTCAGAG | 340 | 323 |
| TufA_371_L | TAATACGACTCACTATAGGGAGAGCACTATAGTAAGGAATATAGC | GCATGTTAGCAGCAGCCCGGGCAGCAGGTGATTGCAGCGGTCAGAG | 169 | 152 |
| InfA | TAATACGACTCACTATAGGCGCAGAGTTGGTTACGCTC | GCATGTCACAGTAACCACGTGACCG | 361 | 344 |
| InfA_L | TAATACGACTCACTATAGGCGCAGAGTTGGTTACGCTC | GCATGTCAGCAGCAGCCCGGGCAGCACAGTAACCACGTGACCG | 387 | 370 |
| Rnr | TAATACGACTCACTATAGGCGATTTGGTTGAAGAGAATCAACC | GCATGTTAGCAGCAGCCCGGGCAGCACGGTTTTTCACGTTTGGTTAAATGTTCG | 235 | 218 |
| Rnr_Tr | TAATACGACTCACTATAGGCATCAGAGATGACAACGGAGG | GCATGTTAGCAGCAGCCCGGGCAGCACGGTTTTTCACGTTTGGTTAAATGTTCG | 173 | 156 |
| tktB | TAATACGACTCACTATAGGAGAAAAACTGTCTGAAGGCATTCG | GCATGTTAGCAGCAGCCCGGGCAGCAATCAGCCATGCCCATCGGCGC | 245 | 227 |
| tktB_Tr | TAATACGACTCACTATAGGCAAACTATAAACCAGCCACGG | GCATGTTAGCAGCAGCCCGGGCAGCAATCAGCCATGCCCATCGGCGC | 176 | 158 |
| MF1 | cgaatttaatacgactcactatagg | GCTTGCATGCCTGCAGACGCA | 121 | 93 |
