# Supplementary material for: How the initiating ribosome copes with ppGpp to translate mRNAs
Source: PLoS Biol. 2020 Jan 29;18(1):e3000593. doi: 10.1371/journal.pbio.3000593 (PMC7010297; doi:10.1371/journal.pbio.3000593)
Supplement: S2 Table — Gene sequences used for mRNA synthesis. A T7 promoter was added by PCR for all natural mRNAs, while mMF1 encoded the promoter in the plasmid. Blue sequences indicate 5′ UTR. Yellow sequences indicate the coding region. Lowercase indicates the start codon. Underlined sequences indicate priming regions for PCR amplifications (S1 Table). A PCR amplification of the template p022 generates a short coding region for in vitro transcription, here called mMF1. T7, bacteriophage T7. (DOCX) [file pbio.3000593.s010.docx]

**S2 Table**

Gene sequences used for mRNA synthesis. A T7 promoter replaced added by PCR for all natural mRNAs while mMF1 encoded the promoter in the plasmid. Blue sequences indicate 5’ untranslated regions (UTR). Yellow sequences indicate the coding region. Lower case indicates the start codon. Underlined sequences indicate priming regions for PCR amplifications (Table S1). A PCR amplification of the template p022 generates a short coding region for *in vitro* transcription, here called mMF1.

| Name | DNA sequences |
| --- | --- |
| *Tuf*A | AAGAAAGCGAAACCAGTTCTGCTTGAGCCGATCATGAAGGTTGAAGTAGAAACTCCGGAAGAGAACACCGGTGACGTTATCGGTGACTTGAGCCGTCGTCGTGGTATGCTCAAAGGTCAGGAATCTGAAGTTACTGGCGTTAAGATCCACGCTGAAGTACCGCTGTCTGAAATGTTCGGATACGCAACTCAGCTGCGTTCTCTGACCAAAGGTCGTGCATCATACACTATGGAATTCCTGAAGTATGATGAAGCGCCGAGTAACGTTGCTCAGGCCGTAATTGAAGCCCGTGGTAAATAAGCCTAAGGGTTAATACCAAAGTCCCGTGCTCTCTCCTGAAGGGGAGAGCACTATAGTAAGGAATATAGCCgtgTCTAAAGAAAAATTTGAACGTACAAAACCGCACGTTAACGTTGGTACTATCGGCCACGTTGACCACGGTAAAACTACTCTGACCGCTGCAATCACCACCGTACTGGCTAAAACCTACGGCGGTGCTGCTCGTGCATTCGACCAGATCGATAACGCGCCGGAAGAAAAAGCTCGTGGTATCACCATCAACACTTCTCACGTTGAATACGACACCCCGACCCGTCACTACGCACACGTAGACTGCCCGGGGCACGCCGACTATGTTAAAAACATGATCACCGGTGCTGCTCAGATGGACGGCGCGATCCTGGTAGTTGCTGCGACTGACGGCCCGATGCCGCAGACTCGTGAGCACATCCTGCTGGGTCGTCAGGTAGGCGTTCCGTACATCATCGTGTTCCTGAACAAATGCGACATGGTTGATGACGAAGAGCTGCTGGAACTGGTTGAAATGGAAGTTCGTGAACTTCTGTCTCAGTACGACTTCCCGGGCGACGACACTCCGATCGTTCGTGGTTCTGCTCTGAAAGCGCTGGAAGGCGACGCAGAGTGGGAAGCGAAAATCCTGGAACTGGCTGGCTTCCTGGATTCTTATATTCCGGAACCAGAGCGTGCGATTGACAAGCCGTTCCTGCTGCCGATCGAAGACGTATTCTCCATCTCCGGTCGTGGTACCGTTGTTACCGGTCGTGTAGAACGCGGTATCATCAAAGTTGGTGAAGAAGTTGAAATCGTTGGTATCAAAGAGACTCAGAAGTCTACCTGTACTGGCGTTGAAATGTTCCGCAAACTGCTGGACGAAGGCCGTGCTGGTGAGAACGTAGGTGTTCTGCTGCGTGGTATCAAACGTGAAGAAATCGAACGTGGTCAGGTACTGGCTAAGCCGGGCACCATCAAGCCGCACACCAAGTTCGAATCTGAAGTGTACATTCTGTCCAAAGATGAAGGCGGCCGTCATACTCCGTTCTTCAAAGGCTACCGTCCGCAGTTCTACTTCCGTACTACTGACGTGACTGGTACCATCGAACTGCCGGAAGGCGTAGAGATGGTAATGCCGGGCGACAACATCAAAATGGTTGTTACCCTGATCCACCCGATCGCGATGGACGACGGTCTGCGTTTCGCAATCCGTGAAGGCGGCCGTACCGTTGGCGCGGGCGTTGTTGCTAAAGTTCTGGGCTAA |
| *Inf*A | CCGCTGCCGATAAGGAATTTTTCGCGTCAGGTAACGCCCATCGTTTATCTCACCGCTCCCTTATACGTTGCGCTTTTGGTGCGGCTTAGCCGTGTGTTTTCGGAGTAATGTGCCGAACCTGTTTGTTGCGATTTAGCGCGCAAATCTTTACTTATTTACAGAACTTCGGCATTATCTTGCCGGTTCAAATTACGGTAGTGATACCCCAGAGGATTAGatgGCCAAAGAAGACAATATTGAAATGCAAGGTACCGTTCTTGAAACGTTGCCTAATACCATGTTCCGCGTAGAGTTAGAAACGGTCACGTGGTTACTGACACACATCTCCGGTAAAATGCGCAAAAACTACATCCGCATCCTGACGGGCGACAAAGTGACTGTTGAACTGACCCCGTACGACCTGAGCAAAGGCCGCATTGTCTTCCGTAGTCGCTGA |
| *Rnr* | CGATTTGGTTGAAGAGAATCAACCGCTTTATAAATTATTGCTGGTGGAGTGACGAAAATCTTCATCAGAGATGACAACGGAGGAACCGAGatgTCACAAGATCCTTTCCAGGAACGCGAAGCTGAAAAATACGCGAATCCCATCCCTAGTCGGGAATTTATCCTCGAACATTTAACCAAACGTGAAAAACCGGCCAGCCGTGATGAGCTGGCGGTAGAACTGCACATTGAAGGCGAAGAGCAGCTTGAAGGCCTGCGTCGCCGCCTGCGCGCGATGGAGCGCGATGGTCAACTGGTCTTCACTCGTCGTCAGTGCTATGCGCTGCCGGAACGCCTCGACCTGGTGAAAGGTACCGTTATTGGCCACCGTGATGGCTACGGCTTTCTGCGGGTTGAAGGGCGTAAAGATGATTTGTATCTCTCCAGCGAGCAGATGAAAACCTGCATTCATGGCGATCAGGTGCTGGCTCAGCCGCTGGGTGCTGACCGTAAAGGTCGTCGTGAAGCGCGTATTGTCCGCGTACTGGTGCCAAAAACCAGCCAGATTGTTGGTCGCTACTTTACCGAAGCGGGCGTCGGCTTTGTGGTTCCTGACGACAGCCGTCTGAGCTTCGATATCTTAATCCCGCCCGATCAGATCATGGGCGCGCGGATGGGCTTTGTGGTCGTAGTCGAACTGACTCAGCGTCCGACTCGCCGCACCAAAGCGGTGGGTAAAATCGTCGAAGTGCTGGGCGACAATATGGGCACCGGCATGGCGGTTGATATCGCTCTGCGTACCCATGAAATTCCGTACATCTGGCCGCAGGCTGTTGAGCAACAGGTTGCCGGGCTGAAAGAAGAAGTGCCGGAAGAAGCAAAAGCGGGCCGTGTTGATCTGCGCGATTTACCGCTGGTCACCATTGATGGCGAAGACGCCCGTGACTTTGACGATGCAGTTTACTGCGAGAAAAAACGCGGCGGCGGCTGGCGTTTATGGGTCGCGATTGCCGACGTCAGCTACTATGTGCGTCCGTCAACGCCGCTGGACAGAGAAGCGCGTAACCGTGGCACGTCGGTGTACTTCCCTTCGCAGGTTATCCCGATGCTGCCGGAAGTGCTCTCTAACGGCCTGTGTTCGCTCAACCCGCAGGTAGACCGCCTGTGTATGGTGTGCGAGATGACGGTTTCGTCGAAAGGCCGCCTGACGGGCTACAAATTCTACGAAGCGGTGATGAGCTCTCACGCGCGTCTGACCTACACCAAAGTCTGGCATATTCTGCAGGGCGATCAGGATCTGCGCGAGCAGTACGCCCCGCTGGTTAAGCATCTCGAAGAGTTGCATAACCTCTATAAAGTGCTGGATAAAGCCCGTGAAGAACGCGGTGGGATCTCATTTGAGAGCGAAGAAGCGAAGTTCATTTTCAACGCTGAACGCCGTATTGAACGTATCGAACAGACCCAGCGTAACGACGCGCACAAATTAATTGAAGAGTGCATGATTCTGGCGAATATCTCGGCGGCGCGTTTCGTTGAGAAAGCGAAAGAACCGGCACTGTTCCGTATTCACGACAAGCCGAGCACCGAAGCGATTACCTCTTTCCGTTCAGTGCTGGCGGAGCTGGGGCTGGAACTGCCGGGCGGTAACAAGCCGGAACCGCGTGACTACGCGGAGCTGCTGGAGTCGGTTGCCGATCGTCCTGATGCAGAAATGCTGCAAACCATGCTGCTGCGCTCGATGAAACAGGCGATTTACGATCCAGAAAACCGTGGTCACTTTGGCCTGGCATTGCAGTCCTATGCGCACTTTACTTCGCCGATTCGTCGTTATCCAGACCTGACGCTGCACCGCGCCATTAAATATCTGCTGGCGAAAGAGCAGGGGCATCAGGGCAACACCACTGAAACCGGCGGCTACCATTATTCGATGGAAGAGATGCTGCAACTGGGTCAGCACTGTTCGATGGCGGAACGTCGTGCCGACGAAGCAACGCGCGATGTGGCTGACTGGCTGAAGTGTGACTTCATGCTCGACCAGGTAGGTAACGTCTTTAAAGGCGTAATTTCCAGCGTCACTGGCTTTGGCTTCTTCGTCCGTCTGGACGACTTGTTCATTGATGGTCTGGTCCATGTCTCTTCGCTGGACAATGACTACTATCGCTTTGACCAGGTAGGGCAACGCCTGATGGGGGAATCCAGCGGCCAGACTTATCGCCTGGGCGATCGCGTGGAAGTTCGCGTCGAAGCGGTTAATATGGACGAGCGCAAAATCGACTTTAGCCTGATCTCCAGCGAACGCGCACCGCGCAACGTCGGTAAAACGGCGCGCGAGAAAGCGAAAAAAGGCGATGCAGGTAAAAAAGGCGGCAAGCGTCGTCAGGTCGGTAAAAAGGTAAACTTTGAGCCAGACAGCGCCTTCCGCGGTGAGAAAAAAACGAAGCCGAAAGCGGCGAAGAAAGACGCGAGAAAAGCGAAAAAGCCATCGGCGAAAACGCAGAAAATAGCTGCAGCGACCAAAGCGAAGCGTGCGGCGAAGAAAAAAGTGGCAGAGTGA |
| *Rnr_Tr* | CATCAGAGATGACAACGGAGGAACCGAGatgTCACAAGATCCTTTCCAGGAACGCGAAGCTGAAAAATACGCGAATCCCATCCCTAGTCGGGAATTTATCCTCGAACATTTAACCAAACGTGAAAAACCGGCCAGCCGTGATGAGCTGGCGGTAGAACTGCACATTGAAGGCGAAGAGCAGCTTGAAGGCCTGCGTCGCCGCCTGCGCGCGATGGAGCGCGATGGTCAACTGGTCTTCACTCGTCGTCAGTGCTATGCGCTGCCGGAACGCCTCGACCTGGTGAAAGGTACCGTTATTGGCCACCGTGATGGCTACGGCTTTCTGCGGGTTGAAGGGCGTAAAGATGATTTGTATCTCTCCAGCGAGCAGATGAAAACCTGCATTCATGGCGATCAGGTGCTGGCTCAGCCGCTGGGTGCTGACCGTAAAGGTCGTCGTGAAGCGCGTATTGTCCGCGTACTGGTGCCAAAAACCAGCCAGATTGTTGGTCGCTACTTTACCGAAGCGGGCGTCGGCTTTGTGGTTCCTGACGACAGCCGTCTGAGCTTCGATATCTTAATCCCGCCCGATCAGATCATGGGCGCGCGGATGGGCTTTGTGGTCGTAGTCGAACTGACTCAGCGTCCGACTCGCCGCACCAAAGCGGTGGGTAAAATCGTCGAAGTGCTGGGCGACAATATGGGCACCGGCATGGCGGTTGATATCGCTCTGCGTACCCATGAAATTCCGTACATCTGGCCGCAGGCTGTTGAGCAACAGGTTGCCGGGCTGAAAGAAGAAGTGCCGGAAGAAGCAAAAGCGGGCCGTGTTGATCTGCGCGATTTACCGCTGGTCACCATTGATGGCGAAGACGCCCGTGACTTTGACGATGCAGTTTACTGCGAGAAAAAACGCGGCGGCGGCTGGCGTTTATGGGTCGCGATTGCCGACGTCAGCTACTATGTGCGTCCGTCAACGCCGCTGGACAGAGAAGCGCGTAACCGTGGCACGTCGGTGTACTTCCCTTCGCAGGTTATCCCGATGCTGCCGGAAGTGCTCTCTAACGGCCTGTGTTCGCTCAACCCGCAGGTAGACCGCCTGTGTATGGTGTGCGAGATGACGGTTTCGTCGAAAGGCCGCCTGACGGGCTACAAATTCTACGAAGCGGTGATGAGCTCTCACGCGCGTCTGACCTACACCAAAGTCTGGCATATTCTGCAGGGCGATCAGGATCTGCGCGAGCAGTACGCCCCGCTGGTTAAGCATCTCGAAGAGTTGCATAACCTCTATAAAGTGCTGGATAAAGCCCGTGAAGAACGCGGTGGGATCTCATTTGAGAGCGAAGAAGCGAAGTTCATTTTCAACGCTGAACGCCGTATTGAACGTATCGAACAGACCCAGCGTAACGACGCGCACAAATTAATTGAAGAGTGCATGATTCTGGCGAATATCTCGGCGGCGCGTTTCGTTGAGAAAGCGAAAGAACCGGCACTGTTCCGTATTCACGACAAGCCGAGCACCGAAGCGATTACCTCTTTCCGTTCAGTGCTGGCGGAGCTGGGGCTGGAACTGCCGGGCGGTAACAAGCCGGAACCGCGTGACTACGCGGAGCTGCTGGAGTCGGTTGCCGATCGTCCTGATGCAGAAATGCTGCAAACCATGCTGCTGCGCTCGATGAAACAGGCGATTTACGATCCAGAAAACCGTGGTCACTTTGGCCTGGCATTGCAGTCCTATGCGCACTTTACTTCGCCGATTCGTCGTTATCCAGACCTGACGCTGCACCGCGCCATTAAATATCTGCTGGCGAAAGAGCAGGGGCATCAGGGCAACACCACTGAAACCGGCGGCTACCATTATTCGATGGAAGAGATGCTGCAACTGGGTCAGCACTGTTCGATGGCGGAACGTCGTGCCGACGAAGCAACGCGCGATGTGGCTGACTGGCTGAAGTGTGACTTCATGCTCGACCAGGTAGGTAACGTCTTTAAAGGCGTAATTTCCAGCGTCACTGGCTTTGGCTTCTTCGTCCGTCTGGACGACTTGTTCATTGATGGTCTGGTCCATGTCTCTTCGCTGGACAATGACTACTATCGCTTTGACCAGGTAGGGCAACGCCTGATGGGGGAATCCAGCGGCCAGACTTATCGCCTGGGCGATCGCGTGGAAGTTCGCGTCGAAGCGGTTAATATGGACGAGCGCAAAATCGACTTTAGCCTGATCTCCAGCGAACGCGCACCGCGCAACGTCGGTAAAACGGCGCGCGAGAAAGCGAAAAAAGGCGATGCAGGTAAAAAAGGCGGCAAGCGTCGTCAGGTCGGTAAAAAGGTAAACTTTGAGCCAGACAGCGCCTTCCGCGGTGAGAAAAAAACGAAGCCGAAAGCGGCGAAGAAAGACGCGAGAAAAGCGAAAAAGCCATCGGCGAAAACGCAGAAAATAGCTGCAGCGACCAAAGCGAAGCGTGCGGCGAAGAAAAAAGTGGCAGAGTGA |
| *tktB* | AGAAAAACTGTCTGAAGGCATTCGTCTGTTCGCCGTTGATCAACGCAAACTGGAAGATCTTCTTGCCGCCAAACTATAAACCAGCCACGGAGTGTTATatgTCCCGAAAAGACCTTGCCAATGCGATTCGCGCACTCAGTATGGATGCGGTACAAAAAGCCAACTCTGGTCATCCCGGCGCGCCGATGGGCATGGCTGATATTGCCGAAGTGCTGTGGAACGATTTTCTTAAACATAACCCTACCGACCCAACCTGGTATGATCGCGACCGCTTTATTCTTTCCAACGGTCACGCGTCGATGCTGCTCTACAGTTTGCTACATCTGACCGGTTACGACCTGCCGCTGGAAGAACTGAAGAACTTCCGTCAGTTGCATTCGAAAACCCCAGGCCACCCGGAGATTGGCTATACGCCAGGCGTTGAAACCACCACCGGCCCGCTTGGACAAGGTTTGGCGAACGCCGTCGGGCTGGCGATAGCAGAGCGTACACTGGCGGCGCAGTTTAACCAGCCAGACCATGAGATCGTCGATCACTTCACCTATGTGTTTATGGGCGACGGCTGCCTGATGGAAGGTATTTCCCACGAAGTCTGTTCGCTGGCAGGCACGCTGGGACTGGGCAAGCTGATTGGTTTTTACGATCACAACGGTATTTCCATCGACGGTGAAACAGAAGGCTGGTTTACCGACGATACGGCAAAACGTTTTGAAGCCTATCACTGGCATGTGATCCATGAAATCGACGGTCACGATCCGCAGGCGGTGAAGGAAGCGATCCTTGAAGCGCAAAGCGTGAAAGATAAGCCGTCGCTGATTATCTGCCGTACGGTGATTGGCTTTGGTTCGCCGAATAAAGCAGGTAAGGAAGAGGCGCACGGCGCACCACTGGGGGAAGAAGAAGTGGCGCTGGCACGGCAAAAACTGGGCTGGCACCATCCGCCATTTGAGATCCCTAAAGAGATTTATCACGCCTGGGATGCCCGTGAAAAAGGCGAAAAAGCGCAGCAGAGCTGGAATGAGAAGTTTGCCGCCTATAAAAAGGCTCATCCGCAACTGGCAGAAGAGTTTACCCGACGGATGAGCGGTGGTTTACCGAAGGACTGGGAGAAAACGACTCAGAAATATATCAATGAGTTACAGGCAAATCCGGCGAAAATCGCTACCCGTAAGGCTTCGCAAAATACGCTTAACGCTTACGGGCCGATGCTGCCTGAGTTGCTCGGCGGTTCGGCGGATCTGGCTCCCAGCAACCTGACCATCTGGAAAGGTTCTGTTTCGCTGAAGGAAGATCCAGCGGGCAACTACATTCACTACGGGGTGCGTGAATTTGGCATGACCGCTATCGCCAACGGCATCGCGCACCACGGCGGCTTTGTGCCGTATACCGCGACGTTCCTGATGTTTGTTGAATACGCCCGTAACGCCGCGCGGATGGCGGCACTGATGAAAGCGCGGCAGATTATGGTTTATACCCACGACTCAATTGGCCTGGGCGAAGATGGTCCGACGCACCAGGCTGTTGAGCAACTGGCCAGCCTGCGCTTAACGCCAAATTTCAGCACCTGGCGACCGTGCGATCAGGTGGAAGCGGCGGTGGGCTGGAAGCTGGCGGTTGAGCGCCACAACGGACCGACGGCACTGATCCTCTCAAGGCAGAATCTGGCCCAGGTGGAACGTACGCCGGATCAGGTTAAAGAGATTGCTCGTGGCGGTTATGTGCTGAAAGACAGCGGCGGTAAGCCAGATATTATTCTGATTGCCACCGGTTCAGAGATGGAAATTACCCTGCAAGCGGCAGAGAAATTAGCAGGAGAAGGTCGCAATGTACGCGTAGTTTCCCTGCCCTCGACCGATATTTTCGACGCCCAGGATGAGGAATATCGGGAGTCGGTGTTGCCTTCTAACGTTGCGGCTCGCGTGGCGGTGGAAGCAGGTATTGCCGATTACTGGTACAAGTATGTTGGTCTGAAAGGGGCAATTGTCGGGATGACGGGTTACGGGGAATCTGCTCCGGCGGATAAGCTGTTCCCGTTCTTTGGCTTTACCGCCGAGAATATTGTGGCAAAAGCGCATAAGGTGCTGGGAGTGAAAGGTGCCTGA |
| *tktB_Tr* | CAAACTATAAACCAGCCACGGAGTGTTATatgTCCCGAAAAGACCTTGCCAATGCGATTCGCGCACTCAGTATGGATGCGGTACAAAAAGCCAACTCTGGTCATCCCGGCGCGCCGATGGGCATGGCTGATATTGCCGAAGTGCTGTGGAACGATTTTCTTAAACATAACCCTACCGACCCAACCTGGTATGATCGCGACCGCTTTATTCTTTCCAACGGTCACGCGTCGATGCTGCTCTACAGTTTGCTACATCTGACCGGTTACGACCTGCCGCTGGAAGAACTGAAGAACTTCCGTCAGTTGCATTCGAAAACCCCAGGCCACCCGGAGATTGGCTATACGCCAGGCGTTGAAACCACCACCGGCCCGCTTGGACAAGGTTTGGCGAACGCCGTCGGGCTGGCGATAGCAGAGCGTACACTGGCGGCGCAGTTTAACCAGCCAGACCATGAGATCGTCGATCACTTCACCTATGTGTTTATGGGCGACGGCTGCCTGATGGAAGGTATTTCCCACGAAGTCTGTTCGCTGGCAGGCACGCTGGGACTGGGCAAGCTGATTGGTTTTTACGATCACAACGGTATTTCCATCGACGGTGAAACAGAAGGCTGGTTTACCGACGATACGGCAAAACGTTTTGAAGCCTATCACTGGCATGTGATCCATGAAATCGACGGTCACGATCCGCAGGCGGTGAAGGAAGCGATCCTTGAAGCGCAAAGCGTGAAAGATAAGCCGTCGCTGATTATCTGCCGTACGGTGATTGGCTTTGGTTCGCCGAATAAAGCAGGTAAGGAAGAGGCGCACGGCGCACCACTGGGGGAAGAAGAAGTGGCGCTGGCACGGCAAAAACTGGGCTGGCACCATCCGCCATTTGAGATCCCTAAAGAGATTTATCACGCCTGGGATGCCCGTGAAAAAGGCGAAAAAGCGCAGCAGAGCTGGAATGAGAAGTTTGCCGCCTATAAAAAGGCTCATCCGCAACTGGCAGAAGAGTTTACCCGACGGATGAGCGGTGGTTTACCGAAGGACTGGGAGAAAACGACTCAGAAATATATCAATGAGTTACAGGCAAATCCGGCGAAAATCGCTACCCGTAAGGCTTCGCAAAATACGCTTAACGCTTACGGGCCGATGCTGCCTGAGTTGCTCGGCGGTTCGGCGGATCTGGCTCCCAGCAACCTGACCATCTGGAAAGGTTCTGTTTCGCTGAAGGAAGATCCAGCGGGCAACTACATTCACTACGGGGTGCGTGAATTTGGCATGACCGCTATCGCCAACGGCATCGCGCACCACGGCGGCTTTGTGCCGTATACCGCGACGTTCCTGATGTTTGTTGAATACGCCCGTAACGCCGCGCGGATGGCGGCACTGATGAAAGCGCGGCAGATTATGGTTTATACCCACGACTCAATTGGCCTGGGCGAAGATGGTCCGACGCACCAGGCTGTTGAGCAACTGGCCAGCCTGCGCTTAACGCCAAATTTCAGCACCTGGCGACCGTGCGATCAGGTGGAAGCGGCGGTGGGCTGGAAGCTGGCGGTTGAGCGCCACAACGGACCGACGGCACTGATCCTCTCAAGGCAGAATCTGGCCCAGGTGGAACGTACGCCGGATCAGGTTAAAGAGATTGCTCGTGGCGGTTATGTGCTGAAAGACAGCGGCGGTAAGCCAGATATTATTCTGATTGCCACCGGTTCAGAGATGGAAATTACCCTGCAAGCGGCAGAGAAATTAGCAGGAGAAGGTCGCAATGTACGCGTAGTTTCCCTGCCCTCGACCGATATTTTCGACGCCCAGGATGAGGAATATCGGGAGTCGGTGTTGCCTTCTAACGTTGCGGCTCGCGTGGCGGTGGAAGCAGGTATTGCCGATTACTGGTACAAGTATGTTGGTCTGAAAGGGGCAATTGTCGGGATGACGGGTTACGGGGAATCTGCTCCGGCGGATAAGCTGTTCCCGTTCTTTGGCTTTACCGCCGAGAATATTGTGGCAAAAGCGCATAAGGTGCTGGGAGTGAAAGGTGCCTGA |
| p022 (MF1) | CGAATTTTAATACGACTCACTATAGGGAATTCAAAAATTTAAAAGTTAACAGGTATACATACT(atg/ttc)TTTACGATTACTACGATCTTCTTCACTTAATGCGTCTGCAGGCATGCAAGCTAAAAAAAAAAAAAAAAAAAAAAAAAAAGCTTGGCACTGGCCGTCGTTTTACAACGTCGTGACTGGGAAAACCCTGGCGTTACCCAACTTAATCGCCTTGCAGCACATCCCCCTTTCGCCAGCTGGCGTAATAGCGAAGAGGCCCGCACCGATCGCCCTTCCCAACAGTTGCGCAGCCTGAATGGCGAATGGAAATTGTAAGCGTTAATATTTTGTTAAAATTCGCGTTAAATTTTTGTTAAATCAGCTCATTTTTTAACCAATAGGCCGAAATCGGCAAAATCCCTTATAAATCAAAAGAATAGACCGAGATAGGGTTGAGTGTTGTTCCAGTTTGGAACAAGAGTCCACTATTAAAGAACGTGGACTCCAACGTCAAAGGGCGAAAAACCGTCTATCAGGGCGATGGCCCACTACGTGAACCATCACCCTAATCAAGTTTTTTGGGGTCGAGGTGCCGTAAAGCACTAAATCGGAACCCTAAAGGGAGCCCCCGATTTAGAGCTTGACGGGGAAAGCCGGCGAACGTGGCGAGAAAGGAAGGGAAGAAAGCGAAAGGAGCGGGCGCTAGGGCGCTGGCAAGTGTAGCGGTCACGCTGCGCGTAACCACCACACCCGCCGCGCTTAATGCGCCGCTACAGGGCGCGTCAGTGGCACTTTTCGGGGAAA |
