# Supplementary material for: How the initiating ribosome copes with ppGpp to translate mRNAs
Source: PLoS Biol. 2020 Jan 29;18(1):e3000593. doi: 10.1371/journal.pbio.3000593 (PMC7010297; doi:10.1371/journal.pbio.3000593)
Supplement: S3 Table — mRNA sequences for MST and in vitro translation (L) analysis. Lowercase indicates the start codon. For mMF1, parenthesis indicate alternative start codons. MST, Microscale Thermophoresis. (DOCX) [file pbio.3000593.s011.docx]

**S3 Table**

mRNA sequences for MST and *in vitro* translation (L) analysis. Lower case indicates the start codon. For mMF1, parenthesis indicate alternative start codons.

| Name | mRNA Sequence |
| --- | --- |
| m*Tuf*A | GGUUCUAUCGCCUUUAAAGAAGGCUUUAAGAAAGCGAAACCAGUUCUGCUUGAGCCGAUCAUGAAGGUUGAAGUAGAAACUCCGGAAGAGAACACCGGUGACGUUAUCGGUGACUUGAGCCGUCGUCGUGGUAUGCUCAAAGGUCAGGAAUCUGAAGUUACUGGCGUUAAGAUCCACGCUGAAGUACCGCUGUCUGAAAUGUUCGGAUACGCAACUCAGCUGCGUUCUCUGACCAAAGGUCGUGCAUCAUACACUAUGGAAUUCCUGAAGUAUGAUGAAGCGCCGAGUAACGUUGCUCAGGCCGUAAUUGAAGCCCGUGGUAAAUAAGCCUAAGGGUUAAUACCAAAGUCCCGUGCUCUCUCCUGAAGGGGAGAGCACUAUAGUAAGGAAUAUAGCCgugUCUAAAGAAAAAUUUGAACGUACAAAACCGCACGUUAACGUUGGUACUAUCGGCCACGUUGACCACGGUAAAACUACUCUGACCGCUGCAAUCACCA |
| m*Tuf*A_L | GGUUCUAUCGCCUUUAAAGAAGGCUUUAAGAAAGCGAAACCAGUUCUGCUUGAGCCGAUCAUGAAGGUUGAAGUAGAAACUCCGGAAGAGAACACCGGUGACGUUAUCGGUGACUUGAGCCGUCGUCGUGGUAUGCUCAAAGGUCAGGAAUCUGAAGUUACUGGCGUUAAGAUCCACGCUGAAGUACCGCUGUCUGAAAUGUUCGGAUACGCAACUCAGCUGCGUUCUCUGACCAAAGGUCGUGCAUCAUACACUAUGGAAUUCCUGAAGUAUGAUGAAGCGCCGAGUAACGUUGCUCAGGCCGUAAUUGAAGCCCGUGGUAAAUAAGCCUAAGGGUUAAUACCAAAGUCCCGUGCUCUCUCCUGAAGGGGAGAGCACUAUAGUAAGGAAUAUAGCCgugUCUAAAGAAAAAUUUGAACGUACAAAACCGCACGUUAACGUUGGUACUAUCGGCCACGUUGACCACGGUAAAACUACUCUGACCGCUGCAAUCACCAUGCUGCCCGGGCUGCUGCUGACAUGC |
| m*Inf*A | GGCGCAGAGUUGGUUACGCUCAUUACCCCGCUGCCGAUAAGGAAUUUUUCGCGUCAGGUAACGCCCAUCGUUUAUCUCACCGCUCCCUUAUACGUUGCGCUUUUGGUGCGGCUUAGCCGUGUGUUUUCGGAGUAAUGUGCCGAACCUGUUUGUUGCGAUUUAGCGCGCAAAUCUUUACUUAUUUACAGAACUUCGGCAUUAUCUUGCCGGUUCAAAUUACGGUAGUGAUACCCCAGAGGAUUAGaugGCCAAAGAAGACAAUAUUGAAAUGCAAGGUACCGUUCUUGAAACGUUGCCUAAUACCAUGUUCCGCGUAGAGUUAGAAAACGGUCACGUGGUUACUG |
| m*Inf*A_L | GGCGCAGAGUUGGUUACGCUCAUUACCCCGCUGCCGAUAAGGAAUUUUUCGCGUCAGGUAACGCCCAUCGUUUAUCUCACCGCUCCCUUAUACGUUGCGCUUUUGGUGCGGCUUAGCCGUGUGUUUUCGGAGUAAUGUGCCGAACCUGUUUGUUGCGAUUUAGCGCGCAAAUCUUUACUUAUUUACAGAACUUCGGCAUUAUCUUGCCGGUUCAAAUUACGGUAGUGAUACCCCAGAGGAUUAGaugGCCAAAGAAGACAAUAUUGAAAUGCAAGGUACCGUUCUUGAAACGUUGCCUAAUACCAUGUUCCGCGUAGAGUUAGAAAACGGUCACGUGGUUACUGUGCUGCCCGGGCUGCUGCUAACAUGC |
| m*Rnr* | GCGAUUUGGUUGAAGAGAAUCAACCGCUUUAUAAAUUAUUGCUGGUGGAGUGACGAAAAUCUUCAUCAGAGAUGACAACGGAGGAACCGAGaugUCACAAGAUCCUUUCCAGGAACGCGAAGCUGAAAAAUACGCGAAUCCCAUCCCUAGUCGGGAAUUUAUCCUCGAACAUUUAACCAAACGUGAAAAACCGUGCUGCCCGGGCUGCUGCUAACAUG |
| m*Rnr*_Tr | GCAUCAGAGAUGACAACGGAGGAACCGAGaugUCACAAGAUCCUUUCCAGGAACGCGAAGCUGAAAAAUACGCGAAUCCCAUCCCUAGUCGGGAAUUUAUCCUCGAACAUUUAACCAAACGUGAAAAACCGUGCUGCCCGGGCUGCUGCUAACAUG |
| m*Tkt*B | GAGAAAAACUGUCUGAAGGCAUUCGUCUGUUCGCCGUUGAUCAACGCAAACUGGAAGAUCUUCUUGCCGCCAAACUAUAAACCAGCCACGGAGUGUUAUaugUCCCGAAAAGACCUUGCCAAUGCGAUUCGCGCACUCAGUAUGGAUGCGGUACAAAAAGCCAACUCUGGUCAUCCCGGCGCGCCGAUGGGCAUGGCUGAUUGCUGCCCGGGCUGCUGCUAACAUGC |
| m*Tkt*B_Tr | GCAAACUAUAAACCAGCCACGGAGUGUUAUaugUCCCGAAAAGACCUUGCCAAUGCGAUUCGCGCACUCAGUAUGGAUGCGGUACAAAAAGCCAACUCUGGUCAUCCCGGCGCGCCGAUGGGCAUGGCUGAUUGCUGCCCGGGCUGCUGCUAACAUGC |
| mMF1 | GGGAAUUCAAAAAUUUAAAAGUUAACAGGUAUACAUACU(aug/uuc)UUUACGAUUACUACGAUCUUCUUCACUUAAUGCGUCUGCAGGCAUGCAAGCgggaauucaaaaauuuaaaaguuaacagguauacauacu |
